# Drug Therapies and Incident Heart Failure in Atrial Fibrillation: A Systematic Review

**DOI:** 10.3390/jcm15208118

**Published:** 2026-10-19

**Authors:** Amirreza Zobdeh, Gregory M. Peterson, Woldesellassie M. Bezabhe, Pankti Shastri, Daniel J. Hoyle

**Affiliations:** School of Pharmacy and Pharmacology, University of Tasmania, Hobart, TAS 7005, Australia; g.peterson@utas.edu.au (G.M.P.); woldesellassie.bezabhe@utas.edu.au (W.M.B.); pankti.shastri@outlook.com (P.S.); daniel.hoyle@utas.edu.au (D.J.H.)

**Keywords:** new-onset heart failure, atrial fibrillation, prevention, pharmacological therapy

## Abstract

**Background/Objectives**: Atrial fibrillation (AF), the most common cardiac arrhythmia, significantly elevates the risk of new-onset heart failure (HF). We aimed to systematically map and appraise available evidence on drug therapies and incident HF in patients with AF. **Methods**: We searched Medline, Embase, Cochrane Central Register of Controlled Trials, and Scopus up to 16 September 2026 for relevant randomized controlled trials (RCTs), sub-analyses of RCTs, and observational/cohort studies. **Results**: Seventeen studies (397,991 participants) were included. The only randomized signal came from a prespecified subgroup of the ACTIVE-I trial, in which irbesartan was associated with fewer first HF hospitalizations than the placebo (HR 0.81, 95% CI 0.69–0.96; moderate certainty). Observational studies showed an association with lower first HF hospitalization for sodium-glucose cotransporter 2 (SGLT2) inhibitors in patients with type 2 diabetes (pooled HR 0.68, 95% CI 0.48–0.95; two studies; low certainty) and lower incident HF diagnosis in a larger cohort that included patients without diabetes (HR 0.86, 95% CI 0.82–0.89). The remaining cohort evidence largely reflected prescribing patterns rather than drug effects. Statins showed an inconsistent association. The higher HF incidence among users of diuretics, mineralocorticoid receptor antagonists, digoxin and renin-angiotensin system inhibitors was consistent with confounding by indication (very low certainty). **Conclusions**: Evidence on drug therapies to prevent HF in AF is limited to one randomized subgroup signal for irbesartan. The remaining findings were associations rather than demonstrated treatment effects. High-quality research, including RCTs in broader AF populations, is needed. PROSPERO Registration: CRD42024587926.

## 1. Introduction

Atrial fibrillation (AF) represents the most prevalent cardiac arrhythmia [1] and is a risk factor for multimorbidity [2]. Its incidence is projected to rise with the aging population, becoming a major public health concern [3]. AF imposes a substantial economic burden on healthcare systems worldwide, with direct costs ranging from US$2000 to US$60,000 per patient per year [4]. Among AF-related comorbidities, heart failure (HF) is particularly prevalent. Individuals with AF have an almost five-fold elevated risk of developing HF [5], and a recent study reports a 40% lifetime risk of new-onset HF following AF diagnosis [6]. HF attributable to AF and atrial flutter has risen more than four-fold globally, from about 163,000 prevalent cases in 1990 to 714,000 in 2021, with 1.3 million cases projected by 2040 [7]. Rapid ventricular rates, irregular rhythm, and the accompanying myocardial inflammatory response drive cardiac remodeling and HF development [8,9,10].

Coexistence of AF and HF significantly worsens outcomes [11]. Their dual presence imposes a substantial burden on the cardiovascular system, leading to frequent hospitalizations, reduced quality of life, and increased mortality [11,12,13,14]. The 2024 European Society of Cardiology (ESC) guidelines on AF employ a systematic framework for AF management called AF-CARE. AF-CARE highlights four key pillars: “C” for comorbidity and risk factor management, “A” for avoiding stroke and thromboembolism, “R” for reducing symptoms by rate and rhythm control, and “E” for evaluation and dynamic reassessment [15]. As highlighted by the first pillar of the AF-CARE pathway, developing effective strategies to prevent comorbidities, such as HF, is critically important, not only to improve survival rates but also to alleviate the significant healthcare burden associated with managing these two intertwined conditions.

Lifestyle modifications, such as physical exercise and smoking cessation, remain the strongest recommendation to prevent HF in patients with AF [16]. The 2023 American Heart Association/American College of Cardiology (AHA/ACC) joint committee AF guidelines made moderate-level recommendations on the potential benefits of rhythm control compared to rate control in preventing cardiac structural abnormalities [16]. The 2022 consensus statement on the current pharmacological prevention and management of heart failure advocates for sodium-glucose cotransporter 2 (SGLT2) inhibitors in patients with type 2 diabetes mellitus (T2DM) and multiple cardiovascular risk factors to reduce HF incidence [17]. Although this recommendation is not specific to AF, it may be applicable to patients with overlapping AF and T2DM.

A narrative review has proposed roles for cardiovascular or cardioprotective drug therapies, such as renin-angiotensin system (RAS) inhibitors, beta-blockers, statins and direct oral anticoagulants (DOACs), while noting that the literature on this topic is limited [18]. Additionally, the results from individual studies are inconsistent and influenced by sample size. For instance, studies on the effects of non-dihydropyridine calcium channel blockers have yielded conflicting results; some suggested a benefit while others found no significant association or differing effects based on patient sex [19,20,21].

Despite the high incidence of new-onset HF in patients with AF, clinicians remain uncertain as to whether commonly prescribed pharmacological therapies modify patients’ trajectory toward HF. Patients with AF are frequently treated with multiple cardiovascular drugs over prolonged periods—yet none of these therapies carries a formal indication for HF prevention in this population, and the evidence base is fragmented and inconsistent. Synthesizing this evidence is a necessary first step toward identifying which, if any, of these agents may reduce HF risk, and where the gaps in clinical knowledge remain most consequential. Therefore, we conducted a systematic review and meta-analysis of studies examining drug therapies associated with the risk of incident HF in patients with AF.

## 2. Materials and Methods

This systematic review was conducted in accordance with Cochrane methodology [22] and reported according to the Preferred Reporting Items for Systematic Reviews and Meta-Analyses (PRISMA) guidelines [23]. The completed PRISMA checklist is provided as Supplementary Material. The review protocol was registered with the International Prospective Register of Systematic Reviews (PROSPERO: CRD42024587926).

### 2.1. Eligibility Criteria

The outcome of interest for this review was new-onset HF, defined as the first diagnosis of HF or first hospital admission for HF. Studies were included if they reported the relationship between the incidence of new-onset HF in patients with AF and a specific drug therapy compared to other drug therapies, placebo, or no treatment. Eligible studies included randomized controlled trials (RCTs) of therapeutic interventions, sub-analyses of RCTs, or observational studies.

Studies were excluded if they were not published in English, included only patients with a prior history of HF, or failed to separately report results for patients with and without a history of HF. Additional exclusion criteria were studies involving participants under 18 years of age, case reports, conference abstracts, dissertations, editorials, opinion pieces, reviews, or research protocols.

### 2.2. Information Sources and Search Strategy

We searched the following major databases: Medline, Embase, Cochrane Central Register of Controlled Trials, and Scopus. The following Population, Intervention, Comparison, Outcome (PICO) framework was used to produce the search terms: Population: patients with AF without previous history of HF; Intervention: drug therapies; Comparison: other drug therapies, placebo, or no treatment; and Outcome: new-onset HF, defined by first hospitalization or first diagnosis of HF. From the PICO, the two main concepts of population (patients with AF) and outcome (new-onset/prevention/risk of HF) were used to develop the database search terms. As it was not possible to encompass all drug therapies using search terms, screening was used to filter the studies with the predefined intervention and comparator. The search terms were reviewed by a University of Tasmania health science librarian prior to execution and are provided in Supplementary Table S1. Searches were conducted from the inception of each database to 29 September 2025. An updated search was subsequently conducted to 16 September 2026 to capture recently published evidence. Additionally, Google Scholar was searched using two prespecified phrases: “drugs to reduce risk of heart failure in AF” and “drugs to prevent heart failure in AF”. The first 200 results returned for each phrase were exported using Publish or Perish software Version is 8.19.5300.9483 [24].

### 2.3. Study Selection, Data Extraction, and Quality Assessment

Using the web-based systematic review platform, Covidence, duplicate articles were automatically removed. In the original search, title screening was performed by a single reviewer (AZ), followed by independent abstract and full-text screening by two reviewers (AZ, DH/WB/GP/PS). For the updated search conducted to 16 September 2026, all newly identified records were screened independently by two reviewers at the title and abstract stage. The reviewers used the pre-specified inclusion/exclusion criteria and noted the reason for the exclusion of any full-text articles. Conflicts were resolved by discussion with a third reviewer. Data were extracted into a standardized form that was piloted on three studies before use; extraction was performed by AZ, and every extracted effect estimate, comparator definition, outcome definition and covariate set was independently verified against the source publication by a second reviewer (DH). Quality assessment was completed by two reviewers independently (AZ, DH/WB/GP). Conflicts in quality assessment were resolved by discussion between the two reviewers.

The revised Cochrane risk-of-bias tool for randomized trials (RoB 2) [25] was used to assess the quality and potential bias across RCTs. The Joanna Briggs Institute (JBI) critical appraisal tools [26] were utilized to assess both methodological quality and potential bias of cohort studies. The JBI appraisal tool for cohort studies consists of an eleven-point checklist to systematically assess the internal validity of research studies. The tool comprehensively evaluates cohort comparability, the reliability of exposure and outcome measurements, the rigorous identification and management of potential confounding factors, the adequacy of follow-up duration, the mitigation of attrition bias, and the appropriateness of the statistical methods employed [26]. The JBI checklist provides four response options for each criterion: Yes, No, Unclear, and Not Applicable. Wherever the JBI appraisal tool refers to the study outcome, we used the pre-specified outcome of this review (new-onset HF) to assess if each study was appropriate to this review’s question.

In addition, the following information from each study was extracted, subject to availability: authors, country, year of publication, number of patients included/excluded and criteria, intervention and control treatments, follow-up period, and patient characteristics—age, sex, comorbidities, type of AF, annual risk of stroke or thromboembolic events (e.g., the Congestive heart failure, Hypertension, Age ≥ 75 years, Diabetes mellitus, Stroke, Vascular disease, Age 65–74 years, and Sex category (CHA_2_DS_2_-VASc) score [27]), and mean heart rate.

### 2.4. Qualitative Synthesis and Meta-Analysis

Hazard ratios (HRs) were used as the preferred effect measure for each intervention. Where available, propensity-matched cohorts or confounder-adjusted HRs through multivariable regression were utilized in preference to univariable regressions. If a study reported only event numbers and no hazard ratios, event numbers were used to calculate risk ratios. Interventions that were assessed in two or more studies were presented in a forest plot. Where study designs and outcomes were of sufficient homogeneity, we performed a meta-analysis. Statistical heterogeneity was examined using the I^2^ statistic and Cochran’s Q test (χ^2^) [28]. Where a meta-analysis consisted of more than two studies, a leave-one-out sensitivity analysis was performed [29]. Meta-analysis was performed using the “R package meta” (version 8.5-0) in the statistical software “R” (version 4.4.1). The random effect was estimated using the method of restricted maximum likelihood (REML) [30]. To account for the small number of studies, the Hartung-Knapp (HK) adjustment was applied to meta-analyses [31]. Where more than two studies were included in a meta-analysis, a prediction interval was calculated [32]. Furthermore, we assessed the certainty of the body of evidence using the Grading of Recommendations Assessment, Development, and Evaluation (GRADEpro) tool (https://gdt.gradepro.org), based on study limitations, inconsistency of effect, imprecision, indirectness, and publication bias. Certainty of the evidence was rated at the following four levels: high, moderate, low, and very low [33].

## 3. Results

### 3.1. Overview of Included Studies

**Figure 1:**
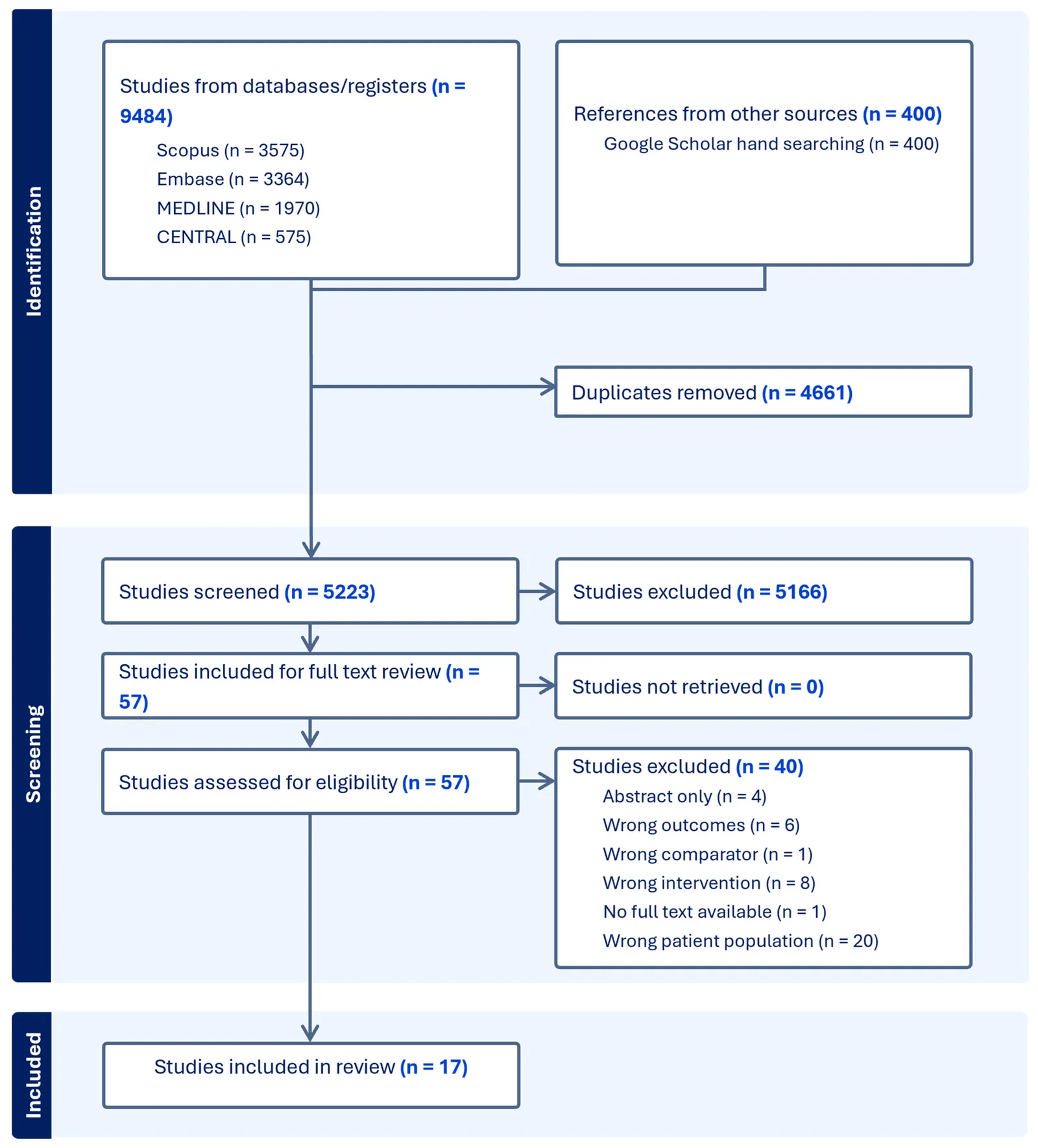
PRISMA flow diagram of the study selection process.

Our search strategy identified 9884 records, of which 4661 duplicate records were removed. Subsequently, 5166 records were excluded due to a lack of relevance at the title and abstract screening step. A total of 57 articles were included in the full-text review. Forty papers were excluded as they had only an abstract (*n* = 4) [34,35,36,37], wrong outcomes (*n* = 6) [38,39,40,41,42,43], wrong comparator (*n* = 1) [44], wrong intervention (*n* = 8) [45,46,47,48,49,50,51,52], wrong patient population (*n* = 20) [53,54,55,56,57,58,59,60,61,62,63,64,65,66,67,68,69,70,71,72] or no full-text available (*n* = 1) [73]. There were 17 original research articles involving a total of 397,991 participants that met our eligibility criteria [19,20,21,74,75,76,77,78,79,80,81,82,83,84,85,86,87]. This included one RCT [85] and 16 cohort studies [19,20,21,74,75,76,77,78,79,80,81,82,83,84,86,87]. Figure 1 outlines the study selection process, and Table 1 provides a summary of the included studies.

### 3.2. Quality Assessment

Risk of bias across all domains for the included RCT was low (Supplementary Figure S1). Most of the observational studies satisfied the majority of JBI criteria. The main recurring limitations related to measurement and control for confounders and the use of routinely collected administrative data for exposure and outcome ascertainment. The assessment is summarized in Supplementary Table S2. Thirteen studies directly examined the association of drug therapies with incident HF in patients with AF [20,21,74,76,77,79,80,81,83,84,85,86,87] and four studies reported on a range of risk factors for HF in patients with AF, including drug therapies [19,75,78,82]. Immortal time bias was not a general feature of the included studies. Except for one cohort [20], most studies defined exposure entirely before time zero [21,74,75,78,81,82,83,84] or used landmarking [19,76,77,79,86,87] or a statistical method [80] to address immortal time bias.

### 3.3. Outcomes

Incident HF was the primary outcome in 8 studies [19,20,21,75,77,78,81,82] and a co-primary or secondary outcome in 9 studies [74,76,79,80,83,84,85,86,87]. Of the 16 cohort studies, 12 used International Classification of Diseases (ICD) codes from a medical database to identify new HF diagnoses and exclude patients with a previous history of HF [19,20,21,76,77,79,80,81,83,84,86,87]. While most of these studies focused solely on first HF hospitalizations, four included outpatient ICD codes [20,81,84,86]. Krisai et al. and Eisen et al. similarly reported the first HF hospitalizations but relied on cohorts extracted from clinical trial data [74,78]. Only two studies, Potpara et al. and Fan et al., used comprehensive assessments, including physical examinations, diagnostic procedures and laboratory results, to detect new HF diagnoses [75,82]. ACTIVE-I trial ascertained the first HF hospitalization as per the trial protocol and confirmed by a blinded event adjudication committee [85].

The definition of HF varied across the studies. The majority analyzed a composite outcome, combining patients with both HF with reduced ejection fraction (HFrEF) and HF with preserved ejection fraction (HFpEF), and did not report separately on each subtype. In contrast, the study by Fan et al. exclusively investigated the HFpEF subtype [75]. A summary of study outcomes, interventions and hazard ratios is reported in Table 2. Studies reporting crude event numbers and calculated risk ratios are reported in Supplementary Table S3.

### 3.4. Randomized Controlled Trial

#### Irbesartan

The ACTIVE-I trial compared irbesartan to placebo in patients with AF and a high risk of stroke. Participants in the irbesartan arm in this trial received 150 mg once per day for the first 2 weeks and then 300 mg once per day. In the pre-specified subgroup analysis of patients without a history of HF, irbesartan was associated with a significant reduction in HF hospitalization (HR 0.81, 95% CI 0.69–0.96, *p* = 0.01) [85]. The certainty of this finding was determined to be moderate according to the GRADEpro tool (Supplementary Table S4a).

### 3.5. Observational Studies

#### 3.5.1. Statins

**Figure 2:**
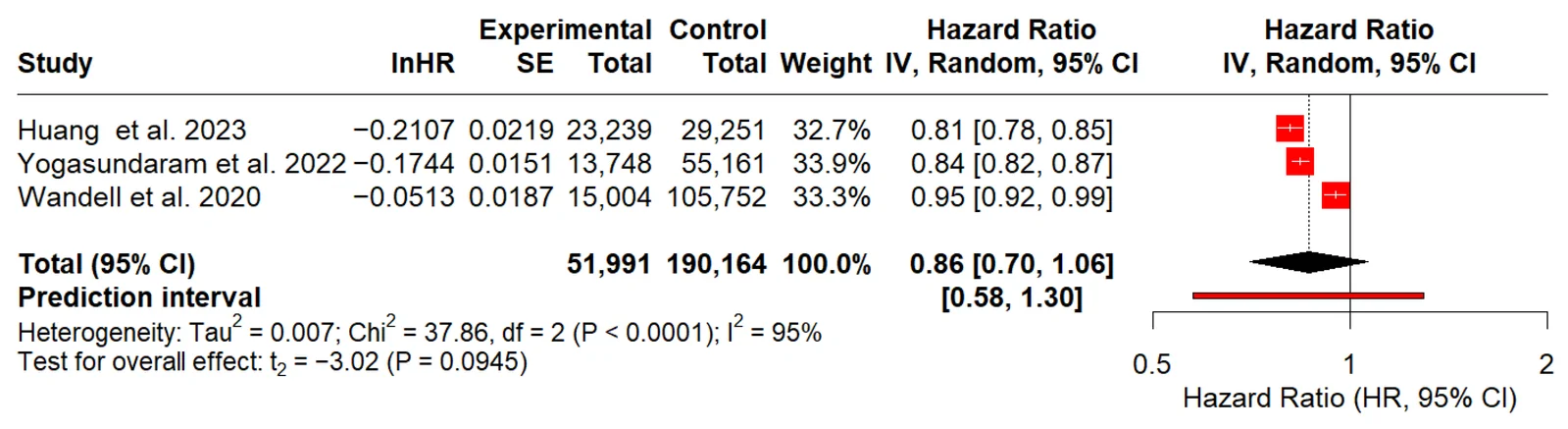
Meta-analysis of statin use and incident heart failure hospitalization in atrial fibrillation [19,21,77]. Ln: Natural logarithm; HR: Hazard ratio; SE: Standard error.

Five studies reported on the association of statin use and new-onset HF [19,20,21,75,77] (Supplementary Figure S2a). While the three studies reporting first HF hospitalization based on administrative coding [19,21,77] each demonstrated that statin use was associated with lower HF hospitalization, a meta-analysis with HK adjustment did not show a statistically significant association (HR 0.86, 95% CI 0.70–1.06; *p* = 0.09; I^2^ = 95%; τ^2^ = 0.007; prediction interval: 0.58–1.30; Figure 2). Similarly, the leave-one-out analysis showed no statistically significant association (Supplementary Figure S4).

The remaining two studies could not be included in the quantitative synthesis as they reported different outcomes. Wandell et al. (2018) did not show a significant association with combined inpatient and outpatient administrative diagnosis of HF (HR 0.91, 95% CI 0.80–1.03) [20]. Fan et al. 2022 reported the number of clinically adjudicated HFpEF in baseline statin users and non-users (RR 0.98, 95% CI 0.55–1.74) (Supplementary Figure S3a) [75]. The certainty of the finding for statins was determined to be very low according to the GRADEpro tool (Supplementary Table S4d–f).

#### 3.5.2. Renin-Angiotensin System Inhibitors

Four studies reported on the association of RAS inhibitors and new-onset HF in patients with AF [19,20,75,82]. As outcomes and measures were different in each study, a meta-analysis was not performed (Supplementary Figure S2b). Yogasundaram et al. reported a slightly higher association in ICD-coded incident HF hospitalization with RAS inhibitor use (HR 1.05, 95% CI 1.02–1.08) [19], whereas Fan et al., in patients with T2DM, found no association with clinically adjudicated HFpEF (HR 0.63, 95% CI 0.37–1.07) [75]. Where only unadjusted event counts were available, RRs were calculated to be 1.08 (95% CI 0.71–1.64) for clinically adjudicated HF [82] and 1.11 (95% CI 1.02–1.21) for ICD-coded HF diagnosis [20] (Supplementary Figure S3b).

When angiotensin-converting enzyme inhibitors (ACEi) and angiotensin receptor blockers (ARB) use was reported separately, both estimates for each came from the Swedish cohorts [20,21] but described different HF endpoints, and were likewise not included in a meta-analysis (Supplementary Figure S2c,d). ACEi use was associated with higher ICD-coded HF hospitalizations (HR 1.24, 95% CI 1.20–1.28) [21] but not with ICD-coded inpatient or outpatient HF diagnosis (HR 1.06, 95% CI 0.95–1.19) [20]. ARB use showed the same pattern (HF hospitalizations HR 1.16, 95% CI 1.11–1.21 [21]; HF diagnosis HR 1.10, 95% CI 0.97–1.26) [20]. The certainty of these findings was determined to be very low according to the GRADEpro tool (Supplementary Table S4g–l).

#### 3.5.3. Sodium-Glucose Cotransporter 2 Inhibitors

**Figure 3:**
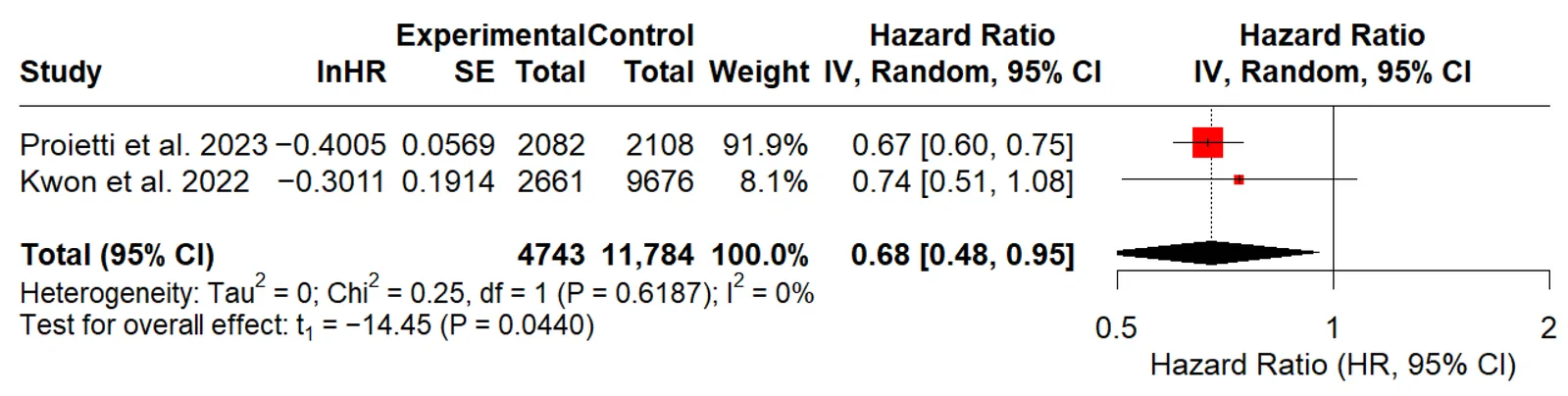
Meta-analysis of sodium-glucose cotransporter 2 inhibitor use and incident heart failure hospitalization in atrial fibrillation [79,83]. Ln: Natural logarithm; HR: Hazard ratio; SE: Standard error.

Three studies reported on the association between the use of SGLT2 inhibitors and new-onset HF [79,83,86]. Fawzy et al. reported an association with lower rates of incident HF diagnosis in 20,457 SGLT2 inhibitor users compared to 20,103 non-users (HR 0.86, 95% CI 0.82–0.89). The propensity-matched study included patients with and without T2DM. Additionally, the authors performed a subgroup analysis in T2DM which showed a similar association with lower incidence of HF (HR 0.82, 95% CI 0.78–0.87) [86]. The other 2 studies reported an association with lower incident HF hospitalization in patients with T2DM in addition to AF [79,83]. Using a REML model with HK adjustment, the meta-analyzed HR for the association between SGLT2 inhibitors and incident HF hospitalization was 0.68 (95% CI 0.48–0.95, *p* = 0.04, I^2^ = 0%, τ^2^ = 0; Figure 3), indicating a significantly lower incidence of first HF hospitalization in users of SGLT2 inhibitors with T2DM (Supplementary Figure S2e). The estimated I^2^ was 0%; however, heterogeneity cannot be estimated reliably from only two studies and this should not be interpreted as evidence that the underlying effects are homogeneous. These findings for SGLT2 inhibitors were determined to be of low certainty according to the GRADEpro tool (Supplementary Table S4b,c).

#### 3.5.4. Rhythm Control Strategy

Yogasundaram et al. did not find an association between antiarrhythmic drug use and first HF hospitalization in AF (HR 0.96, 95% CI 0.93–1.00; *p* = 0.09) [19]. Gao et al. conducted a propensity-matched comparison of dronedarone to other antiarrhythmics and found no significant difference in new-onset HF between dronedarone and sotalol (HR 1.20, 95% CI 0.80–1.80; *p* = 0.39), flecainide (HR 0.80, 95% CI 0.50–1.30; *p* = 0.37) or propafenone (HR 0.9, 95% CI 0.5–1.40; *p* = 0.70) [76]. However, they found an increased incidence of HF in patients taking amiodarone compared to dronedarone (HR 2.80, 95% CI 2.00–3.90; *p* < 0.01) [76]. Kipp et al. found a lower incidence of HF in users of class IC antiarrhythmics compared to class III (HR 0.70, 95% CI 0.64–0.76) [87].

#### 3.5.5. Rate Control Strategy

Westergaard et al. compared each class of rate-control agents to beta-blockers. There was no difference between beta-blockers and non-dihydropyridine calcium channel blockers (CCBs) (HR 1.06, 95% CI 0.94–1.16; *p* = 0.28), but lower rates of new-onset HF were associated with digoxin (HR 0.9, 95% CI 0.85–0.94; *p* < 0.01) and the use of two or more rate control agents (HR 0.81, 95% CI 0.78–0.84; *p* < 0.01) [84].

##### Beta-Blockers

Four studies assessed the association of beta-blocker use and the incidence of new-onset HF [19,20,75,82] (Supplementary Figure S2f). Yogasundaram et al. found no association with ICD-coded HF hospitalizations (HR 1.02, 95% CI 0.99–1.05) [19] and Fan et al. found no association with clinically adjudicated HFpEF in patients with T2DM (HR 0.77, 95% CI 0.45–1.31) [75]. The RRs derived from unadjusted event counts were 0.99 (95% CI 0.61–1.60) for clinically adjudicated HF [82] and 0.70 (95% CI 0.64–0.77) for ICD-coded HF diagnosis [20] (Supplementary Figure S3c).

Two studies reported on beta-1-selective and non-beta-1-selective beta-blockers separately [20,21] (Supplementary Figure S2g,h). Beta-1-selective beta-blocker use was associated with fewer ICD-coded HF hospitalizations (HR 0.92, 95% CI 0.90–0.95) [21] but not with ICD-coded inpatient or outpatient HF diagnosis (HR 0.96, 95% CI 0.86–1.08) [20]. Non-beta-1-selective beta-blockers were associated with lower ICD-coded incident HF hospitalizations (HR 0.93, 95% CI 0.88–0.98) [21] and HF diagnosis (HR 0.84, 95% CI 0.72–0.98) [20]. The certainty of the findings for beta-blockers was determined to be very low according to the GRADEpro tool (Supplementary Table S4m–s).

##### Calcium Channel Blockers

Two studies reported on CCBs as a combination of heart and vessel-active CCBs [20,75] (Supplementary Figure S3d). Neither reported hazard ratios. The RRs calculated were 0.77 (95% CI 0.70–0.85) for ICD-coded HF diagnosis [20] and 1.41 (95% CI 0.76–2.63) for clinically adjudicated HFpEF [75].

Three studies reported on heart-active (non-dihydropyridine) CCBs separately [19,20,21] (Supplementary Figure S2i). While two studies reported ICD-coded incident HF hospitalizations [19,21], they showed opposite associations and statistical heterogeneity between them was too great (I^2^ = 94%) for a summary estimate to be meaningful, so no meta-analysis was performed: Wandell et al. (2020) reported a higher rate of incident HF hospitalization (HR 1.12, 95% CI 1.02–1.22) [21] and Yogasundaram et al. a lower incident HF hospitalization (HR 0.92, 95% CI 0.88–0.95) [19]. The third study reported ICD-coded HF diagnosis and showed no association (HR 0.81, 95% CI 0.64–1.03) [20].

Two studies reported on vessel-active (dihydropyridine) CCBs separately [20,21] (Supplementary Figure S2j), reporting an HR of 0.76 (95% CI 0.67–0.86) for ICD-coded HF diagnosis [20] and HR 1.06 (95% CI 1.01–1.1) for ICD-coded HF hospitalizations [21]. The certainty of the findings for CCBs was determined to be very low according to the GRADEpro tool (Supplementary Table S4t–y).

##### Digoxin

Three studies assessed the association of digoxin with new-onset HF hospitalization [19,20,74] (Supplementary Figure S2k). Wandell et al. (2018) found no association with ICD-coded inpatient or outpatient HF diagnosis (HR 1.03, 95% CI 0.92–1.15) [20], and Eisen et al., in a propensity-matched trial cohort, found no association with HF hospitalizations (HR 1.18, 95% CI 0.91–1.51) [74]. Yogasundaram et al. reported a substantially higher rate of ICD-coded incident HF hospitalizations in digoxin users (HR 1.50, 95% CI 1.45–1.55) [19]. The certainty of this finding was determined to be very low according to the GRADEpro tool (Supplementary Table S4z–aa).

#### 3.5.6. Diuretics

Three studies assessed the association of diuretics with the development of new-onset HF [19,20,82] (Supplementary Figure S2l). Yogasundaram et al. reported a markedly higher rate of ICD-coded incident HF hospitalizations in users of diuretics (HR 3.05, 95% CI 2.97–3.15) [19]. The RRs derived from unadjusted event counts were 1.30 (95% CI 0.82–2.07) for clinically adjudicated HF [82] and 1.55 (95% CI 1.41–1.72) for ICD-coded HF diagnosis Supplementary Figure S3e [20].

Two studies reported on loop diuretics, thiazides and mineralocorticoid inhibitors [20,21]. Wandell et al. (2020) reported ICD-coded HF hospitalizations [21] while Wandell et al. (2018) reported ICD-coded inpatient or outpatient HF diagnosis [20]. Loop diuretic use was associated with a higher occurrence of incident HF (HF diagnosis HR 1.41, 95% CI 1.25–1.57; HF hospitalizations HR 1.28, 95% CI 1.23–1.34) (Supplementary Figure S2m), as was mineralocorticoid receptor antagonist use (HF diagnosis HR 1.22, 95% CI 1.04–1.44; HF hospitalizations HR 1.39, 95% CI 1.28–1.50) (Supplementary Figure S2n). Thiazide use was associated with a lower rate of ICD-coded HF diagnosis (HR 0.74, 95% CI 0.65–0.84) but showed no association with HF hospitalizations (HR 0.98, 95% CI 0.93–1.03) (Supplementary Figure S2o). The certainty of these findings was determined to be very low according to the GRADEpro tool (Supplementary Table S4bb–ii).

#### 3.5.7. Oral Anticoagulants

Three studies assessed the association of oral anticoagulant (OAC) therapy with the development of new-onset HF [19,75,82] (Supplementary Figure S2p). Yogasundaram et al. found no association with ICD-coded HF hospitalizations (HR 1.00, 95% CI 0.97–1.03) [19]; the RRs derived from unadjusted event counts were 1.25 (95% CI 0.76–2.04) for clinically adjudicated HF [82] and 1.12 (95% CI 0.59–2.11) for clinically adjudicated HFpEF [75] (Supplementary Figure S3f). Lin et al. compared different OACs to one another, finding DOACs were associated with lower rates of incident HF compared to warfarin in patients with T2DM and AF (HR 0.8, 95% CI 0.74–0.86; *p* < 0.01) [81]. Lee et al. reported higher number of first HF hospitalization in patients receiving warfarin (RR 1.23, 95% CI 0.98–1.55) [80]. The certainty of this finding was determined to be very low according to the GRADEpro tool (Supplementary Table S4jj–ll).

## 4. Discussion

To our knowledge, this is the first systematic review to consolidate, across drug classes, the evidence linking pharmacological therapy to the risk of new-onset HF in patients with AF. The evidence base is thin and, with a single exception, observational. Only one included study was an RCT. This study assessed an intervention added to standard of care for preventive intent rather than a therapy prescribed for another indication and assessed for its incidental association with HF. In the pre-specified subgroup of ACTIVE-I without a history of HF, irbesartan was associated with fewer HF hospitalizations than placebo (HR 0.81, 95% CI 0.69–0.96) with moderate certainty [85]. All other estimates in this review were derived from cohort studies and were rated low (SGLT2 inhibitors) or very low (all remaining comparisons) in certainty. The associations observed for SGLT2 inhibitors are directionally consistent and biologically plausible, but they are associations, not demonstrated treatment effects. Equally, the higher HF incidence observed among users of diuretics, mineralocorticoid inhibitors, digoxin and RAS inhibitors should not be read as evidence that these drugs cause HF. For most of the drug classes examined here, the plausible explanation for the observed associations is who received the drug, not the direct effect of the drug.

### 4.1. Heart Failure Phenotypes

All but one study failed to distinguish HFrEF from HFpEF. The distinction between HFrEF and HFpEF is a substantive gap, as these subtypes have distinct cellular, causal, and treatment pathways [88]. A drug that prevents one and not the other would be expected to show a diluted or null association against a composite endpoint. Future cohorts and trials in this population should report incident HF by phenotype, using echocardiographic and natriuretic peptide data rather than diagnostic coding alone.

### 4.2. Renin-Angiotensin System Inhibitors

Beyond lowering blood pressure, RAS inhibitors reduce HF risk in other at-risk populations, including patients with hypertension or prior myocardial infarction [89,90]. The ACTIVE-I trial, an RCT, showed that irbesartan reduced the risk of incident HF hospitalization compared to placebo in patients with AF and high risk factors for stroke [85]. First HF hospitalization in participants without prior HF was a pre-specified subgroup within the trial and HF hospitalization was confirmed by a blinded event adjudication committee. Generalizability of these results to other RAS inhibitors and the broader AF population with varying thromboembolic risk profiles remains unknown.

In contrast, three of the major retrospective cohort studies showed higher rates of incident HF hospitalization among patients using RAS inhibitors [19,20,21]. This contrast is most likely due to confounding by indication. RAS inhibitors are used to treat HF, so inadequately excluded pre-existing HF can be misclassified as new-onset. Patients prescribed them are also at higher baseline cardiovascular risk, and thus more prone to HF. The study by Yogasundaram et al. had only a one-year follow-up post-AF diagnosis [19], which may have been too short to detect a treatment effect, since progression to new-onset HF in AF is gradual. Furthermore, the indications for RAS inhibitor initiation might inherently predispose patients to a greater HF risk, necessitating careful adjustment in analyses. Similarly, the study by Wandell et al. (2018) reported a higher event rate of incident HF among users of RAS inhibitors [20]. However, the adjusted HRs reported separately for ACEis and ARBs were not statistically significant [20]. While the later-published study by Wandell et al. (2020) accounted for comorbidities and had an average follow-up of 5.3 years, the study authors posited that the elevated HF incidence in RAS inhibitor users may have been due to residual confounding by indication or competing risks [21]. Because HF history relied on hospitalization records alone, community-managed HF patients started on RAS inhibitors may have been included, biasing the association.

### 4.3. Statins

The three cohort studies reporting first HF hospitalizations from administrative coding each showed an association with lower incidence of HF hospitalizations. However, once meta-analyzed, with Hartung-Knapp adjustment applied, the pooled estimate was not statistically significant (HR 0.86, 95% CI 0.70–1.06; I^2^ = 95%; τ^2^ = 0.007) [19,21,77]. Despite the large number of participants, this evidence rests on only three studies, which differed substantially in methodology. The high statistical heterogeneity and subsequently the large confidence interval post Hartung-Knapp adjustment is driven by Wandell et al. 2020 [21] study. Wandell et al. (2020) [21] followed patients with established AF for incident HF, up to nine years after AF diagnosis, whereas Yogasundaram et al. and Huang et al. assessed cohorts with newly diagnosed AF. Exposure definitions ranged from a single baseline prescription (Wandell et al.) to at least 90 days of continuous use within the first year after diagnosis (Huang et al.). The very low GRADE certainty of evidence reflects the inconsistency in effect size and risk of bias arising from the observational nature of these studies. The smaller study reporting combined inpatient and outpatient HF diagnosis showed no statistically significant association [20].

One feature of the statin data deserves comment. Statins are indicated for atherosclerotic cardiovascular disease (ASCVD) and dyslipidemia, both of which independently raise HF risk, so confounding by indication would be expected to bias this association toward harm rather than benefit—which is the opposite of what was observed in each of the observational studies. However, healthy adherer effects and residual confounding by socioeconomic and health-seeking factors remain plausible. Randomized data from populations outside AF are consistent with a modest reduction in HF events with statin therapy [91]; nonetheless, whether statins confer an additional benefit in AF specifically, independent of cholesterol level and prior atherosclerotic disease, cannot be resolved from these data and would require a randomized comparison.

### 4.4. SGLT2 Inhibitors

The two propensity-matched studies of SGLT2 inhibitors showed an association with lower incidence of HF hospitalization in patients with AF and T2DM (HR 0.68, 95% CI 0.48–0.95; I^2^ = 0%, τ^2^ = 0). The low GRADE certainty stems from two non-randomized studies with limited follow-up (2.1 and 3 years) that failed to adjust for glycemic control, leaving findings vulnerable to residual confounding by diabetes severity and duration. A third, larger propensity-matched cohort of new users, which included patients with and without T2DM and ascertained HF from inpatient and outpatient diagnoses, reported a smaller but directionally consistent association (HR 0.86, 95% CI 0.82–0.89), with a similar estimate in its T2DM subgroup. As its outcome definition differed, it was not pooled [86]. There is mechanistic coherence with the established cardiorenal benefits of SGLT2 inhibition [17,92,93,94,95]. This is complemented by recent randomized evidence suggesting broader electrophysiological effects. Specifically, a meta-analysis of eight adjudicated trials (N = 58,569) identified a significant reduction in sudden cardiac death associated with empagliflozin and dapagliflozin (OR 0.82, 95% CI 0.72–0.94; I^2^ = 0%) [96]. AF and HF also share inflammatory pathways, including activation of the NLRP3 inflammasome and pro-fibrotic signaling from epicardial adipose tissue, that promote atrial structural remodeling, and experimental data suggest that SGLT2 inhibition may attenuate this inflammatory signaling [97]. However, definitive establishment of SGLT2 inhibition as a preventive pathway in AF requires direct randomized data evaluating incident HF in populations beyond T2DM.

### 4.5. Rhythm and Rate Control Strategies

The irregular rhythm and rapid heart rate caused by AF contribute to the development of HF [8,9,10]. Symptom control is an important pillar of managing AF [15,16]. Despite this, the body of literature evaluating association between rate and/or rhythm control strategies and incidence of HF is limited. Among the included studies, the higher HF incidence among amiodarone users compared to dronedarone reported by Gao et al., using administrative codes, may be explained by a short 1.5-year follow-up period, a brief 6-month window of prior clinical data to flag pre-existing HF and contraindications for dronedarone in severe HF, which inherently biases sicker patients toward amiodarone [76]. Likewise, the lower HF incidence with class IC than class III antiarrhythmic drugs reported by Kipp et al. [87] should be interpreted with some caution. Despite the propensity-based methods with falsification endpoints and appropriate exclusion of prior HF or myocardial infarction, echocardiographic data were unavailable to the authors; thus, factors such as structural heart disease, which would steer prescribing away from class IC agents, may have persisted.

Similarly, findings regarding rate-control agents show notable discrepancies. Westergaard et al. reported a lower association of HF incidence with digoxin and two or more rate-control agents compared to beta-blockers, a finding likely confounded by the higher overall mortality observed in the digoxin and polytherapy groups [84]. While Yogasundaram et al. noted a higher association of ICD-coded incident HF hospitalizations among digoxin users, this is attributable to confounding by indication (digoxin being preferentially prescribed in pre-existing HF), a brief 1-year follow-up, and reliance on administrative codes [19]. In contrast, Wandell et al. (2018) found no association using combined inpatient and outpatient HF diagnoses [20], and Eisen et al. reported null findings in a propensity-matched trial cohort [74]. The potential for immortal time bias in the study by Wandell et al. 2018 [20] means lower association with beta-blockers and CCBs should be interpreted with caution, as this would tend to favor treated patients. Evidence surrounding beta-blockers and CCBs remains similarly inconclusive due to the inherent limitations of observational designs.

Trial-level data also present interpretative challenges. A subgroup analysis of the AFFIRM trial by Slee et al. [43] compared rate-control with rhythm-control strategies in a subgroup of patients without a previous history of HF. The study defined HF emergence as an increase of 2 or more in the New York Heart Association (NYHA) functional classification score. While demonstrating a higher incidence in the rate-control group (HR 1.32, 95% CI 1.04–1.69), the metric reflects functional decline rather than a definitive clinical diagnosis of HF, and the study was consequently excluded from our analysis. Nevertheless, current 2023 AHA/ACC AF guidelines cite this AFFIRM sub-analysis as moderate-level evidence supporting the potential benefits of rhythm control over rate control in preventing adverse cardiac structural remodeling [16].

Although clinical paradigms have shifted toward early rhythm control, the precise impact on the incidence of de novo HF remains unclear. In EAST-AFNET 4 [98], early rhythm control compared to standard of care in patients with recently diagnosed AF and cardiovascular comorbidity reduced a composite of cardiovascular death, stroke and hospitalizations for worsening HF or acute coronary syndrome. However, HF-related hospitalization was evaluated as part of a composite endpoint rather than a dedicated incident-HF endpoint, and approximately 25% of participants had prevalent HF at baseline. Consequently, whether early, structured rhythm control genuinely prevents incident HF in patients with AF remains an open question and, given the biological plausibility of tachycardia-mediated remodeling, represents a high-priority area for future research.

Although outside the pharmacological scope of this review, catheter ablation has emerged as a first-line rhythm-control option in selected patients [16], and in a propensity-matched cohort it was associated with a lower risk of incident HF than antiarrhythmic drugs [99]. Additionally paired with advanced techniques such as cardiac magnetic resonance, atrial cardiomyopathy can be identified before it potentially progresses to advanced atrial failure and clinical heart failure [100]. Thus, future pharmacological studies of HF prevention in AF should include ablation as a strong covariate in their analysis.

### 4.6. Oral Anticoagulants

While the included studies did not demonstrate a significant association between oral anticoagulant use and incident HF [19,75,82], these findings must be interpreted independently of the well-established role of anticoagulation in stroke prevention, which is supported by robust randomized evidence. Comparisons between specific agents warrant similar caution. Lin et al. reported lower HF incidence with DOACs than warfarin among patients with AF and T2DM [81]. These findings may be susceptible to residual confounding arising from renal function, frailty, bleeding risk, adherence and comorbidity burden, all of which influence the choice between warfarin and DOACs. Anticoagulation decisions should therefore continue to be driven by stroke and bleeding risk rather than potential effect on HF. This is illustrated by patients after apparently successful AF ablation, a growing population in whom the need for long-term anticoagulation is uncertain [101]. An updated meta-analysis of 267,443 patients found that discontinuing OACs after apparently successful ablation was associated with a lower risk of major bleeding without a statistically significant increase in thromboembolic events [102]. Such cohorts, in which the balance of benefit and harm is genuinely contested, may be an informative setting in which to examine any association between OAC use and incident HF.

### 4.7. Diuretics and Mineralocorticoid Inhibitors

The observed associations between diuretics, mineralocorticoid inhibitors and an increased HF incidence reflect underlying clinical phenotypes, confounding by indication and incomplete exclusion of pre-existing HF rather than direct causal toxicity. Their initiation could serve as a marker for patients already traversing the early stages of the HF trajectory. Given that randomized evidence demonstrates a mortality and morbidity benefit of mineralocorticoid inhibitors in established HF [103] and loop diuretics are an important part of symptom management in established HF [104], these findings should not be interpreted as drug-related harm, and clinicians should not withhold these therapies where they are indicated. They do, however, highlight the critical need to distinguish preventive therapies from marker medications in observational research.

### 4.8. Limitations

The literature on the pharmacological prevention of HF in AF is relatively limited. Although this is the first consolidation of evidence across drug classes for this indication, individual drug groups were often supported by only a few studies. Furthermore, heterogeneity across studies regarding effect measures, comparators, and outcome definitions precluded quantitative pooling for most comparisons, necessitating a narrative synthesis of the findings.

The outcome of interest, new-onset HF, was itself clinically heterogeneous. Included studies ascertained first HF hospitalization, inpatient or outpatient ICD-coded HF diagnosis, clinically adjudicated HF, or HFpEF specifically. These endpoints differ in sensitivity, specificity and clinical meaning: hospitalization captures more severe and often later presentations, whereas outpatient diagnoses may capture earlier disease. Studies also differed in how completely they excluded pre-existing HF, with several relying on hospital records alone and therefore unable to exclude community-managed HF, and in how exposure was defined (baseline use, new initiation or landmarked exposure). We therefore pooled only studies sharing an outcome definition and described each result using the endpoint the study reported; findings for one endpoint should not be assumed to apply to the others.

Most observational studies compared drug users to the rest of the cohort (non-users) rather than to a standardized placebo or active control, so pooled estimates should be interpreted cautiously given study-specific design and confounding.

Most studies were observational and retrospective, frequently using hospitalization ICD codes to identify new HF or exclude previous HF. This introduced the inherent risk of confounding by indication, where the indication for a drug therapy may have been independently associated with the development of HF. Cohort studies showed higher HF incidence with medications used to treat HF (diuretics, mineralocorticoid receptor antagonists, digoxin, RAS inhibitors). This should not be interpreted as a marker for toxicity but an indicator of confounding.

As all included studies either omitted AF subtypes or did not analyze them separately, we were unable to stratify our findings by paroxysmal, persistent, or permanent AF. When including multiple AF subtypes, future investigations should incorporate subgroup analyses by each subtype.

The included studies also span a wide calendar period during which the management of AF and related comorbidities has changed substantially. This includes the adoption of DOACs, the introduction of SGLT2 inhibitors and the move toward early rhythm control. Several cohorts predate these changes, so their treated and untreated populations do not correspond to those seen in contemporary practice. Additionally, trends in HF diagnosis and coding may contribute to the observed associations. Comparability with current practice may therefore be limited.

Many cohort studies also did not measure time-on-treatment; baseline medication status may not reflect actual exposure given discontinuation or therapy changes, underscoring the need for time-dependent exposure assessment.

In the original search, title screening was performed by one reviewer. While duplicate screening of all abstracts and full texts limits the likelihood of a relevant record being overlooked, this possibility cannot be excluded.

### 4.9. Implications for Practice, Policy, and Research

The current evidence reinforces the critical role of effective management of underlying comorbidities, such as T2DM and ASCVD, in patients with AF. Consistent with the AHA/ACC and ESC guidelines, clinicians should continuously assess and optimize the management of these comorbidities throughout a patient’s AF journey [15,16].

Further rigorous research is needed. While RCTs are desirable, cohort studies can contribute if they use enhanced methods: drawing on general practice and specialist databases (beyond hospitalization records) for better flagging of HF, accounting for duration of exposure, and applying propensity matching to align intervention and comparator groups.

## 5. Conclusions

This systematic review identified the literature on the association of statins, SGLT2 inhibitors, RAS inhibitors, diuretics, anticoagulants, and rate and rhythm control agents with new-onset HF in patients with AF. The ACTIVE-I subgroup analysis provided the only randomized signal identified, suggesting fewer first HF hospitalizations with irbesartan among patients with AF and elevated stroke risk. Because HF hospitalization was a secondary outcome assessed within a pre-specified subgroup, this finding requires confirmation in trials specifically designed to evaluate incident HF. The role of other therapies remains unclear given the predominance of observational studies using administrative database codes for outcome ascertainment, which carries inherent bias and confounding risks. High-quality research is needed.

## Figures and Tables

**Table 1:** Summary of included studies.

Authors	Cohort Size	Country	Follow-Up Period (Years)	Subpopulation	Outcome	Baseline HF Exclusion	Mean Age	Male Sex, *n*	AF Subtype
Randomized controlled trial									
ACTIVE-I Investigators 2011 [85]	6135	Across 30 countries	4.1	History of risk factors for stroke	First HF hospitalization as per study protocol	Assessed by the site investigator as part of baseline case report form.	Not reported for subgroup without HF at baseline		
Prospective cohort study									
Fan et al. 2022 [75]	552	China	5	Type 2 diabetes mellitus	Incident HF with preserved ejection fraction. Identified by symptoms/signs + LVEF ≥ 50% + elevated natriuretic peptide + Evidence of LV diastolic dysfunction	Baseline echocardiogram, HF symptoms, and cardiomyopathy.	68.1	314	NR
Retrospective cohort study									
Potpara et al. 2013 [82]	842	Serbia	5	Patients with structurally normal hearts	Incident HF diagnosis. Clinically adjudicated by symptoms, physical examination, and ECG	Baseline echocardiogram and Clinical history	51.6	531	Paroxysmal and Persistent
Fawzy et al. 2026 [86]	40,560	UK	3	-	Incident HF inpatient or outpatient diagnosis (ICD coding)	Baseline HF inpatient or outpatient diagnosis (ICD coding)	Not reported for subgroup without HF at baseline		
Lin et al. 2023 [81]	24,708	Taiwan	3	Type 2 diabetes mellitus	Incident HF inpatient or outpatient diagnosis (ICD coding)	Baseline HF inpatient or outpatient diagnosis (ICD coding)	Not reported for subgroup without HF at baseline		
Westergaard et al. 2023 [84]	7408	Denmark	1	-	Incident HF inpatient or outpatient diagnosis (ICD coding)	Baseline HF inpatient or outpatient diagnosis (ICD coding)	78	3395	NR
Wandell et al. 2018 [20]	7975	Sweden	5.7	-	Incident HF inpatient or outpatient diagnosis (ICD coding)	Baseline HF inpatient or outpatient diagnosis (ICD coding)	72.3	4510	NR
Proietti et al. 2023 [83]	4190	UK	3	Type 2 diabetes mellitus	Incident HF hospitalization (ICD coding)	Baseline HF inpatient or outpatient diagnosis (ICD coding)	Not reported for subgroup without HF at baseline		
Kwon et al. 2022 [79]	12,337	Korea	2.1	Type 2 diabetes mellitus	Incident HF hospitalization (ICD coding)	Baseline HF inpatient or outpatient diagnosis (ICD coding)	Not reported for subgroup without HF at baseline		
Lee et al. 2017 [80]	1643	Taiwan	2	Long-term hemodialysis	Incident HF hospitalization (ICD coding)	Baseline HF inpatient or outpatient diagnosis (ICD coding)	Not reported for subgroup without HF at baseline		
Huang et al. 2023 [77]	52,490	Hong Kong	5.1	-	Incident HF hospitalization (ICD coding)	Baseline HF inpatient or outpatient diagnosis (ICD coding)	74.7	27,657	NR
Krisai et al. 2021 [78]	23,503	Canada	2	-	Incident HF hospitalization (Trial cohort)	History of prior HF as per individual trial protocols	71.3	14,582	Paroxysmal, Persistent or Permanent
Eisen et al. 2017 [74]	8981	Across 46 countries	2.8	CHADS_2_ score of 2 or more	First HF hospitalization (Trial cohort)	Baseline history of clinical HF Class C (structural heart disease with prior or current symptoms of heart failure, such as shortness of breath, fatigue, decreased exercise tolerance) or Class D (refractory heart failure requiring specialized interventions)	73	5517	Paroxysmal, Persistent or Permanent
Gao et al. 2013 [76]	6120	USA	1.5	-	Incident HF hospitalization (ICD coding)	Baseline HF inpatient or outpatient diagnosis (ICD coding)	Not reported for subgroup without HF at baseline		
Yogasundaram et al. 2022 [19]	68,909	Canada	1	-	Incident HF hospitalization (ICD coding)	HF hospitalization (ICD coding)	78.6	31,861	NR
Wandell et al. 2020 [21]	120,756	Sweden	5.3	-	Incident HF hospitalization (ICD coding)	HF hospitalization (ICD coding)	74.9	67,656	NR
Kipp et al. 2019 [87]	10,882	USA	4.9	-	Incident HF hospitalization (ICD coding)	Baseline HF inpatient or outpatient diagnosis (ICD coding)	67.3	10,607	NR

AF: Atrial fibrillation; CHADS_2_: Congestive heart failure, Hypertension, Age (75 or older), Diabetes, and Stroke; ECG: Electrocardiogram; HF: Heart failure; ICD: International classification of diseases; LV: Left ventricular; LVEF: Left ventricular ejection fraction; NR: Not reported; UK: United Kingdom; USA: United States of America.

**Table 2:** Summary of study interventions and hazard ratios.

Authors	Intervention (Event/Sample)	Comparator (Event/Sample)	Results: HR (95% CI)	Exposure Definition	Statistical Method (Main Covariates)
**Randomized Controlled Trial (Outcome: Incident HF Hospitalization)**					
ACTIVE-I Investigators 2011 [85]	**Irbesartan** (NR/3058)	**Placebo** (NR/3077)	0.81 (95% CI 0.69–0.96)	Randomized placebo-controlled at baseline	Adjusted (participation in ACTIVE-A or W study)
**Prospective cohort study (Outcome: Incident HF with preserved ejection fraction. Adjudicated by symptoms/signs + LVEF ≥ 50%+ Elevated natriuretic peptide + Evidence of LV diastolic dysfunction)**					
Fan et al. 2022 [75]	**Beta-blockers** (22/237)	**No beta-blockers** (40/315)	0.77 (95% CI 0.45–1.31)	Baseline use	Adjusted (age, gender, COPD, eGFR, LAD, LVMI, E/e’, AF/BP/glycemic control)
	**ACEis/ARBs** (40/398)	**No ACEis/ARBs** (22/154)	0.63 (95% CI 0.37–1.07)		
**Retrospective cohort study (Outcome: Incident HF diagnosis. Adjudicated by Symptoms + Physical examination + ECG)**					
Potpara et al. 2013 [82]	**Aspirin** (49/401)	**No Aspirin** (34/441)	1.90 (95% CI 1.20–3.10)	Baseline use	Unadjusted
**Retrospective cohort study (Outcome: Incident HF inpatient or outpatient diagnosis)**					
Fawzy et al. 2026 [86]	**SGLT2 inhibitors** (3991/20,457)	**No SGLT2 inhibitors** (4750/20103)	0.86 (95% CI 0.82–0.89)	New initiation	PSM (89 socioeconomic, comorbidity, procedure, laboratory and medication covariates)
Lin et al. 2023 [81]	**DOACs** (4512/19,591)	**Warfarin** (1404/5117)	0.80 (95% CI 0.74–0.86)	New initiation	IPTW (40 socioeconomic, comorbidity, medication covariates)
Westergaard et al. 2023 [84]	**≥2 rate control drugs** (NR/1821)	**Beta-blockers** (NR/2893)	0.81 (95% CI 0.78–0.84)	Baseline use	Adjusted (sex, age, ECG year, Anticoagulation, RASi, statins, diabetes, hypertension, ischemic heart disease, kidney disease and stroke)
	**Verapamil or diltiazem** (NR/662)	**Beta-blockers** (NR/2893)	1.06 (95% CI 0.94–1.16)		
	**Digoxin** (NR/2032)	**Beta-blockers** (NR/2893)	0.90 (95% CI 0.85–0.94)		
Wandell et al. 2018 [20]	**Digoxin** (581/2381)	**No Digoxin** (971/5594)	1.03 (95% CI 0.92–1.15)	New initiation	Adjusted (sex, age, educational level, marital status, neighborhood score, myocardial infarction, valvular heart disease, cardiomyopathy, cerebrovascular diseases, hypertension, obesity, diabetes, COPD, obstructive sleep apnea syndrome, depression and anxiety)
	**Thiazides** (387/2454)	**No Thiazides** (1165/5521)	0.74 (95% CI 0.65–0.84)		
	**Loop diuretics** (867/3225)	**No Loop diuretics** (685/4750)	1.41 (95% CI 1.25–1.57)		
	**Aldosterone antagonists** (193/715)	**No Aldosterone antagonists** (1359/7260)	1.22 (95% CI 1.04–1.44)		
	**Beta-1-selective beta-blockers** (962/5544)	**No Beta-1-selective beta-blockers** (590/2431)	0.96 (95% CI 0.86–1.08)		
	**Non-selective beta-blockers** (208/1407)	**No Non-selective beta-blockers** (1344/6568)	0.84 (95% CI 0.72–0.98)		
	**Heart-active CCBs** (78/458)	**No Heart-active CCBs** (1474/7517)	0.81 (95% CI 0.64–1.03)		
	**Vessel-active CCBs** (393/2454)	**No Vessel-active CCBs** (1159/5521)	0.76 (95% CI 0.67–0.86)		
	**ACEis** (558/2924)	**No ACEis** (994/5051)	1.06 (95% CI 0.95–1.19)		
	**ARBs** (349/1966)	**No ARBs** (1203/6009)	1.10 (95% CI 0.97–1.26)		
	**Statins** (440/2883)	**No Statins** (1112/5092)	0.91 (95% CI 0.80–1.03)		
**Retrospective cohort study (Outcome: Incident HF hospitalization)**					
Proietti et al. 2023 [83]	**SGLT2 inhibitors** (496/2082)	**No SGLT2 inhibitors** (736/2108)	0.67 (95% CI 0.60–0.75)	Baseline use	PSM (43 socioeconomic, comorbidity, medication covariates)
Kwon et al. 2022 [79]	**SGLT2 inhibitors** (31/2661)	**No SGLT2 inhibitors** (210/9676)	0.74 (95% CI 0.51–1.08)	New initiation	PSM (31 socioeconomic, comorbidity, medication covariates)
Kipp et al. 2019 [87]	**Class IC antiarrhythmic** (271/3973)	**Class III antiarrhythmic** (884/6909)	0.70 (95% CI 0.64–0.76)	New initiation	IPTW (28 socioeconomic, comorbidity, medication covariates)
Huang et al. 2023 [77]	**Statins** (3673/23,239)	**No Statins** (5595/29,251)	0.81 (95% CI 0.78–0.85)	Baseline use	IPTW (27 socioeconomic, comorbidity, medication, procedure covariates)
Krisai et al. 2021 [78]	**Antiarrhythmic drugs** (count NR)	**No Antiarrhythmic drugs** (count NR)	1.18 (95% CI NR)	Baseline use	Unadjusted
Eisen et al. 2017 [74]	**Digoxin** (80/1825)	**No Digoxin** (287/7156)	1.18 (95% CI 0.91–1.51)	Baseline use	PSM (38 socioeconomic, comorbidity, medication, covariates)
Gao et al. 2013 [76]	**Amiodarone** (count NR)	**Dronedarone** (count NR)	2.80 (95% CI 2.00–3.90)	New initiation	PSM (age, gender, cohort entry year, number of AF/AFL diagnoses, MI, stroke, diabetes, hypertension, dyslipidemia, and ventricular arrhythmia during the baseline period)
	**Sotalol** (count NR)	**Dronedarone** (count NR)	1.20 (95% CI 0.80–1.80)		
	**Flecainide** (count NR)	**Dronedarone** (count NR)	0.80 (95% CI 0.50–1.30)		
	**Propafenone** (count NR)	**Dronedarone** (count NR)	0.90 (95% CI 0.50–1.40)		
Yogasundaram et al. 2022 [19]	**Antiarrhythmics** (NR/7585)	**No Antiarrhythmics** (NR/61,324)	0.96 (95% CI 0.93–1.00)	Baseline use	Adjusted (sex, age, Hospital type, Income, Residential area, diabetes, hypertension, coronary artery disease, peripheral vascular disease, stroke/TIA/systemic embolism, chronic kidney disease, electrical cardioversion)
	**Beta-blockers** (NR/29,838)	**No Beta-blockers** (NR/39,071)	1.02 (95% CI 0.99–1.05)		
	**ACEis/ARBs** (NR/17,040)	**No ACEis/ARBs** (NR/51,869)	1.05 (95% CI 1.02–1.08)		
	**Heart-active CCBs** (NR/8704)	**No Heart-active CCBs** (NR/60,205)	0.92 (95% CI 0.88–0.95)		
	**Digoxin** (NR/8376)	**No Digoxin** (NR/60,533)	1.50 (95% CI 1.45–1.55)		
	**Diuretics** (NR/15,733)	**No Diuretics** (NR/53,176)	3.05 (95% CI 2.97–3.15)		
	**Oral antihyperglycemics** (NR/4770)	**No Oral antihyperglycemics** (NR/64,139)	0.84 (95% CI 0.80–0.88)		
	**Statins** (NR/13,748)	**No Statins** (NR/55,161)	0.84 (95% CI 0.82–0.87)		
	**ADP receptor antagonist** (NR/1610)	**No ADP receptor antagonist** (NR/67,299)	0.89 (95% CI 0.82–0.97)		
	**Oral anticoagulation** (NR/27,360)	**No Oral anticoagulation** (NR/41,549)	1.00 (95% CI 0.97–1.03)		
Wandell et al. 2020 [21]	**Loop diuretics** (2137/7524)	**No Loop diuretics** (26,120/113,232)	1.28 (95% CI 1.23–1.34)	Baseline use	Adjusted (age, region of Sweden, immigrant status, socioeconomic factors, comorbidities, and all drug classes. CHD, cardiomyopathy, and valvular heart diseases)
	**Thiazides** (1455/6004)	**No Thiazides** (26,802/114,752)	0.98 (95% CI 0.93–1.03)		
	**Potassium-saving agents** (686/1992)	**No Potassium-saving agents** (27,571/118,764)	1.39 (95% CI 1.28–1.50)		
	**Beta-1-selective beta-blockers** (7798/35,969)	**No Beta-1-selective beta-blockers** (20,459/84,787)	0.92 (95% CI 0.90–0.95)		
	**Non-selective beta-blockers** (1536/7577)	**No Non-selective beta-blockers** (26,721/113,179)	0.93 (95% CI 0.88–0.98)		
	**Heart-active CCBs** (482/1932)	**No Heart-active CCBs** (27,775/118,824)	1.12 (95% CI 1.02–1.22)		
	**Vessel-active CCBs** (2818/10,444)	**No Vessel-active CCBs** (25,439/110,312)	1.06 (95% CI 1.01–1.10)		
	**ACEis** (4566/15,377)	**No ACEis** (23,691/105,379)	1.24 (95% CI 1.2–1.28)		
	**ARBs** (2039/7197)	**No ARBs** (26,218/113,559)	1.16 (95% CI 1.11–1.21)		
	**Statins** (3714/15,004)	**No Statins** (24,543/105,752)	0.95 (95% CI 0.92–0.99)		

ACEi: angiotensin-converting enzyme inhibitor; ADP: adenosine diphosphate; AF: atrial fibrillation; AFL: atrial flutter; ARB: angiotensin receptor blocker; BP: blood pressure; CCB: calcium channel blocker; CHD: coronary heart disease; CI: confidence interval; COPD: chronic obstructive pulmonary disease; DOAC: direct oral anticoagulant; E/e’: E and E’ waves ratio; ECG: electrocardiogram; eGFR: estimated glomerular filtration rate; HF: heart failure; HR: hazard ratio; IPTW: inverse probability of treatment weighting; LAD: left atrium diameter; LV: left ventricular; LVEF: left ventricular ejection fraction; LVMI: left ventricular mass index; MI: myocardial infarction; NR: not reported; PSM: propensity score matching; RASi: Renin-angiotensin system inhibitors; SGLT2: Sodium-glucose cotransporter 2; TIA: transient ischemic attack.

## Data Availability

The data that support the findings which did not fit within Supplementary Materials of this study are available from the corresponding author upon reasonable request.

## Supplementary Materials

The following supporting information can be downloaded at https://www.mdpi.com/article/10.3390/jcm15208118/s1.

## Abbreviations

The following abbreviations are used in this manuscript:

AF	Atrial fibrillation
ACC	American College of Cardiology
ACEi	Angiotensin-Converting Enzyme inhibitor
AHA	American Heart Association
ARB	Angiotensin II Receptor Blocker
ASCVD	Atherosclerotic Cardiovascular Disease
CCB	Calcium Channel Blocker
CI	Confidence Interval
COPD	Chronic Obstructive Pulmonary Disease
CHA2DS2-VASc	Congestive heart failure, Hypertension, Age ≥75 years, Diabetes mellitus, Stroke, Vascular disease, Age 65–74 years, and Sex category
ESC	European Society of Cardiology
GRADE	Grading of Recommendations Assessment, Development, and Evaluation
HF	Heart failure
HFpEF	Heart failure with preserved ejection fraction
HFrEF	Heart failure with reduced ejection fraction
HK	Hartung-Knapp
HR	Hazard ratio
ICD	International Classification of Diseases
IPTW	Inverse Probability of Treatment Weighting
JBI	Joanna Briggs Institute
MRA	Mineralocorticoid Receptor Antagonist
NYHA	New York Heart Association
DOAC	direct oral anticoagulant
OAC	Oral anticoagulant
PICO	Population, Intervention, Comparison, Outcome
PRISMA	Preferred Reporting Items for Systematic Reviews and Meta-Analyses
RAS	Renin-angiotensin system
RCT	Randomized controlled trial
REML	Restricted maximum likelihood
RoB	Risk of Bias
RR	Risk ratio
SGLT2	Sodium-glucose cotransporter 2
T2DM	Type 2 diabetes mellitus
