# Supplementary material for: Drug Therapies and Incident Heart Failure in Atrial Fibrillation: A Systematic Review
Source: J Clin Med. 2026 Oct 19;15(20):8118. doi: 10.3390/jcm15208118 (PMC13706031; doi:10.3390/jcm15208118)
Supplement: Supplementary file 1 [file jcm-15-08118-s001.zip › Supplemental Publication Material.pdf]

Supplemental Publication Material

Supplementary Table S1: Search strategy and databases

Database	Search strategy	Articles
**Ovid Medline**	*Line Search Term*<br>*1 (atrial* adj2 (fibrillat* or flutter*)).ab,ti,kf.*<br>*2 exp Atrial Flutter/*<br>*3 exp Atrial Fibrillation/*<br>*4 1 or 2 or 3*<br>*5 ((HF or CHF or CCF) adj3 (new* or onset* or risk* or initial* or inciden* or de-novo* or new-onset* or prevent*)).ab,ti,kf.*<br>*6 ((congestive or heart or cardiac) adj (failur* or decompensat* or de-compensate* or incompetence* or insufficienc*) adj3 (new* or onset* or risk* or initial* or inciden* or de-novo* or new-onset* or prevent*)).ab,ti,kf.*<br>*7 5 or 6*<br>*8 exp Animals/ not Humans/*<br>*9 english.lg.*<br>*10 (news or congres* or book* or chapter* or dissertation* or editorial* or Conference abstract*).pt.*<br>*11 exp Review/ or Systematic Review/ or Meta-Analysis/ or Case Reports/*<br>*12 (4 and 7 and 9) not (8 or 10 or 11)*	1970
**Ovid Embase**		3364
**Scopus**	*(*<br>*TITLE-ABS-KEY ((hf OR chf or CCF) W/3 (new* or onset* or risk* or initial* or incident* or de-novo* or new-onset* or prevent*))*<br>*OR TITLE-ABS-KEY (((congestive or heart OR cardiac) W/1 (failur* OR decompensate* OR de-compensate* OR incompetence* OR insufficienc*)) W/3 (new* or onset* or risk* or initial* or incident* or de-novo* or new-onset* or prevent*))*<br>*)*<br>*AND (*<br>*TITLE-ABS-KEY (atrial* W/3 (fibrillat* OR flutter*))*<br>*OR TITLE-ABS-KEY (af OR afib OR aflutter)*<br>*)*<br>*AND NOT (*<br>*(INDEXTERMS(animals OR animal))*<br>*AND NOT (INDEXTERMS(humans OR human))*<br>*)*<br>*AND (*<br>*LIMIT-TO (DOCTYPE,"ar")*<br>*)*<br>*AND (*<br>*LIMIT-TO (LANGUAGE,"English")*<br>*)*	3575
**CENTRAL**	*1 (atri* near/3 (fibrillat* or flutter*)):ti,ab,kw*<br>*2 MeSH descriptor: [Atrial Fibrillation] explode all trees*<br>*3 MeSH descriptor: [Atrial Flutter] explode all trees*<br>*4 #2 OR #1 OR #3*<br>*5 ((new* or onset* or risk* or initial* or inciden* or de NEXT novo* or new NEXT onset* or prevent*) NEAR/3 ((decompensate* OR de NEXT compensate* or congestive or heart OR cardiac) NEXT (failur* OR decompensation* OR incompetence* OR insufficienc*))):ti,ab,kw*<br>*6 ((new* or onset* or risk* or initial* or inciden* or de NEXT novo* or new NEXT onset* or prevent*) NEAR/3 (CHF OR HF or CCF)):ti,ab,kw*<br>*7 #5 OR #6*<br>*8 #4 AND #7*	575
**Google Scholar**	*Hand search of first 200 results: “drugs to reduce risk of heart failure in AF” and “drugs to prevent heart failure in AF”*	400
**Total**		9884

CENTRAL: Cochrane Central Register of Controlled Trials

**Supplementary Table S2: Quality assessment of each study based on The Joanna Briggs Institute (JBI) critical appraisal tool [1]**	Proietti et al. 2023[2]	Yogasundaram et al. 2022[3]	Westergaard et al. 2023[4]	Wandell et al. 2020[5]	Wandell et al. 2018[6]	Lee et al. 2017[7]	Potpara et al. 2013[8]	Gao et al. 2013[9]	Krisai et al. 2021[10]
Were the two groups similar and recruited from the same population?	Unclear	Unclear	Yes	Unclear	Unclear	Unclear	Unclear	Yes	Unclear
Were the exposures measured similarly to assign people to both exposed and unexposed groups	No	Yes	Yes	Yes	Yes	No	Unclear	Yes	Yes
Was the exposure measured in a valid and reliable way?	No	Yes	Yes	No	No	No	No	Yes	Unclear
Were confounding factors identified?	No	Yes	Yes	Yes	Yes	No	Yes	No	Yes
Were strategies to deal with confounding factors stated?	Yes	Yes	Yes	No	No	Yes	No	Yes	Yes
Were the groups/participants free of the outcome at the start of the study?	Yes	Yes	Yes	Yes	Yes	No	Yes	Yes	Unclear
Were the outcomes measured in a valid and reliable way?	Yes	Yes	No	Yes	Yes	No	Yes	No	No
Was the follow up time reported and sufficient to be long enough for outcomes to occur?	Yes	No	No	Yes	Yes	Yes	Yes	No	Yes
Was follow up complete, and if not, were the reasons to loss to follow up described and explored?	Yes	Yes	Unclear	Unclear	Unclear	Unclear	Yes	Unclear	Unclear
Were strategies to address incomplete follow up utilized?	unclear	Yes	Unclear	Unclear	Unclear	Yes	Yes	Not Applicable	Yes
Was appropriate statistical analysis used?	Yes	Yes	Yes	Yes	Yes	Yes	Yes	Yes	Unclear

**Supplementary table S2 continued:**	Huang et al. 2023[11]	Fan et al. 2022[12]	Kwon et al. 2022[13]	Eisen et al. 2017[14]	Lin et al. 2023[15]	Fawzy et al. [16]	Kipp et al. [17]
Were the two groups similar and recruited from the same population?	Unclear	Unclear	Yes	Yes	Yes	Unclear	No
Were the exposures measured similarly to assign people to both exposed and unexposed groups	Yes	Yes	Yes	Yes	Yes	Yes	Yes
Was the exposure measured in a valid and reliable way?	Yes	Yes	No	Yes	Yes	Unclear	Yes
Were confounding factors identified?	Yes	Yes	Yes	Yes	Unclear	Yes	Yes
Were strategies to deal with confounding factors stated?	Yes	Yes	Yes	Yes	Yes	Yes	Yes
Were the groups/participants free of the outcome at the start of the study?	Yes	Yes	Yes	Yes	Yes	Yes	Yes
Were the outcomes measured in a valid and reliable way?	Yes	No	Yes	Yes	Yes	No	No
Was the follow up time reported and sufficient to be long enough for outcomes to occur?	Yes	Yes	Yes	Yes	Yes	Yes	Yes
Was follow up complete, and if not, were the reasons to loss to follow up described and explored?	Yes	Yes	Yes	Unclear	Unclear	No	No
Were strategies to address incomplete follow up utilized?	Not Applicable	Not Applicable	Yes	Unclear	Unclear	Unclear	Unclear
Was appropriate statistical analysis used?	Yes	Yes	Yes	Yes	Yes	Yes	Yes

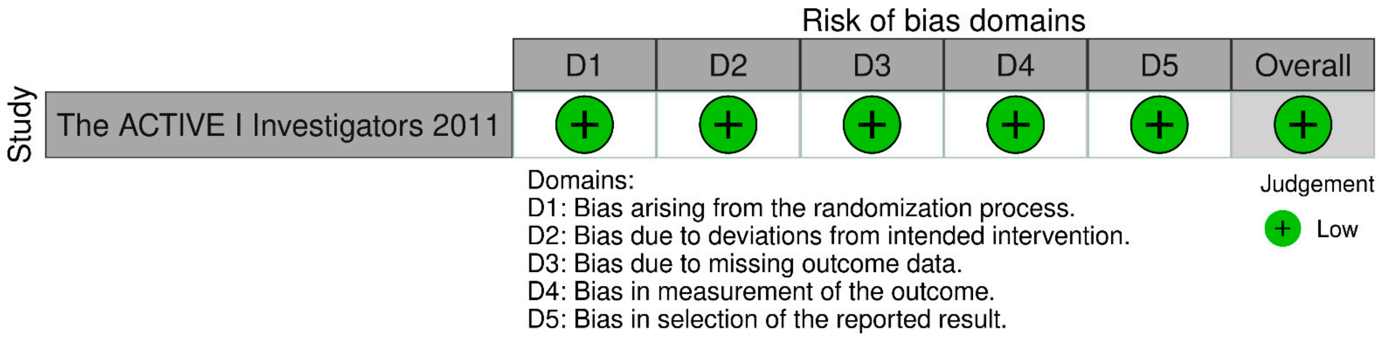

Supplementary Figure S1: The Cochrane risk-of-bias tool for randomized trials (RoB 2) traffic-light plot

Supplementary Table S3: Summary of studies reporting crude incidence for interventions

Authors (number of participants)	Intervention (event/Sample)	Comparator (event/Sample)	Percentages	Calculated risk ratios (95% CI)
**Retrospective cohort study (Outcome: Incident HF diagnosis by Symptoms + Physical examination + ECG)**				
Potpara et al. 2013 [8] (n= 842) *	**Diuretics** (21/174)	**No Diuretics** (62/668)	12.1% vs 9.3%	1.30 (0.82–2.07)
	**Drugs slowing AV conduction** (50/488)	**No Drugs slowing AV conduction** (33/354)	10.2% vs 9.3%	1.10 (0.72–1.67)
	**Beta-blockers** (19/195)	**No Beta-blockers** (64/647)	9.7% vs 9.9%	0.99 (0.61–1.60)
	**Class IA or IC drugs or sotalol** (16/145)	**No Class IA or IC drugs or sotalol** (67/697)	11.0% vs 9.6%	1.15 (0.69–1.92)
	**Amiodarone** (17/121)	**No Amiodarone** (66/721)	14.0% vs 9.2%	1.53 (0.93–2.52)
	**ACEis/ARBs** (32/310)	**No ACEis/ARBs** (51/532)	10.3% vs 9.6%	1.08 (0.71–1.64)
	**Oral anticoagulants** (18/153)	**No Oral anticoagulants** (65/689)	11.8% vs 9.4%	1.25 (0.76–2.04)
**Prospective cohort study (Outcome: Incident HF with preserved ejection fraction. Adjudicated by symptoms/signs + LVEF ≥50%+ Elevated natriuretic peptide + Evidence of LV diastolic dysfunction)**				
Fan et al. 2022 [12] (n= 552) †	**CCB** (51/423)	**No CCB** (11/129)	12.1% vs 8.5%	1.41 (0.76–2.63)
	**Statins** (13/118)	**No Statins** (49/434)	11.0% vs 11.3%	0.98 (0.55–1.74)
	**Oral anticoagulants** (10/81)	**No Oral anticoagulants** (52/471)	12.3% vs 11.0%	1.12 (0.59–2.11)
	**Class Ic/III AADs** (12/137)	**No Class Ic/III AADs** (50/415)	8.8% vs 12.1%	0.73 (0.40–1.32)
	**Sulfonylureas** (26/227)	**No Sulfonylureas** (36/325)	11.5% vs 11.1%	1.03 (0.64–1.66)
	**Glinides** (4/33)	**No Glinides** (58/519)	12.1% vs 11.2%	1.08 (0.42–2.81)
	**α-GIs** (6/60)	**No α-GIs** (56/492)	10.0% vs 11.4%	0.88 (0.40–1.95)
	**Insulins** (15/117)	**No insulins** (47/435)	12.8% vs 10.8%	1.19 (0.69–2.04)
	**DPP-4 inhibitors** (3/23)	**No DPP-4 inhibitors** (59/529)	13.0% vs 11.2%	1.17 (0.40–3.45)
**Retrospective cohort study (Outcome: Incident HF inpatient or outpatient diagnosis)**				
Wandell et al. 2018 [6] (n= 7,975)	**Any diuretic treatment** (1098/4856)	**No diuretic treatment** (454/3119)	22.6% vs 14.6%	1.55 (1.41–1.72)
	**All beta-blockers** (1083/6111)	**No beta-blockers** (469/1864)	17.7% vs 25.2%	0.70 (0.64–0.77)
	**RAS-blocking agents** (800/3901)	**No RAS-blocking agents** (752/4074)	20.5% vs 18.5%	1.11 (1.02–1.21)
	**Calcium channel blockers** (459/2821)	**No Calcium channel blockers** (1093/5154)	16.3% vs 21.2%	0.77 (0.70–0.85)
**Retrospective cohort study (Outcome: Incident HF Hospitalization)**				
Lee et al. 2017 [7]	**Warfarin** (82/403)	**No Warfarin** (205/1240)	20.3% vs 16.5%	1.23 (0.98–1.55)

*Subpopulation of patients with structurally normal hearts

†Subpopulation of patients with type 2 diabetes mellitus

α-GI: alpha-glucosidase inhibitor; AAD: Antiarrhythmic drugs; ACEi: angiotensin-converting enzyme inhibitors; ARB: angiotensin receptor blocker; AV: Atrioventricular; CCB: Calcium channel blocker; DPP-4: Dipeptidyl Peptidase 4; ECG: Electrocardiogram; HF: Heart Failure; RAS: Renin-Angiotensin System;

Supplementary Table S4: GRADE summary of findings.

<table>
<tr><th colspan="7">Certainty assessment</th><th colspan="2">№ of patients (Event/Total)</th><th rowspan="2">Relative Effect (95% CI)</th><th rowspan="2">Certainty</th></tr>
<tr><th>№ of studies</th><th>Study design</th><th>Risk of bias</th><th>Inconsistency</th><th>Indirectness</th><th>Imprecision</th><th>Other considerations</th><th>Intervention</th><th>Comparator</th></tr>
<tr><td colspan="11">a) Irbesartan compared to placebo for prevention of incident heart failure hospitalization in patients with atrial fibrillation and a history of risk factors for stroke (follow-up: 4.1 years)</td></tr>
<tr><td>1 [18]</td><td>Randomised trial</td><td>Not serious</td><td>Not serious</td><td>Not serious</td><td>Serious</td><td>None</td><td>Not reported in this study / 3058</td><td>Not reported in this study / 3077</td><td>HR 0.81
(0.69 to 0.96)</td><td>⊕⊕⊕◯
Moderate</td></tr>
<tr><td colspan="11">b) SGLT2 inhibitors compared to no SGLT2 inhibitors and association with incidence of heart failure hospitalization in patients with atrial fibrillation and Type 2 Diabetes Mellitus (follow-up: range 2.1 to 3 years)</td></tr>
<tr><td>2 [2, 13]</td><td>Non-randomised observational cohort studies</td><td>Not serious
Both propensity score matched</td><td>Not serious
I^2=0%</td><td>Not serious</td><td>Not serious</td><td>None</td><td>527/4743</td><td>946/11784</td><td>HR 0.68
(0.48 to 0.95)</td><td>⊕⊕◯◯
Low</td></tr>
<tr><td colspan="11">c) SGLT2 inhibitors compared to no SGLT2 inhibitors and association with incidence heart failure diagnosis in patients with atrial fibrillation (follow-up: 3 years)</td></tr>
<tr><td>1 [16]</td><td>Non-randomised observational cohort studies</td><td>Not serious
propensity score matched</td><td>Not serious</td><td>Not serious</td><td>Not serious</td><td>None</td><td>3991/20457</td><td>4750/20103</td><td>HR 0.86
(0.82 to 0.89)</td><td>⊕⊕◯◯
Low</td></tr>
</table>

d) Statins compared to no statins and association with incidence of heart failure hospitalization in patients with atrial fibrillation **(follow-up: range 1 to 5.3 years)**

3[3, 5, 11]	Non-randomised observational cohort studies	Serious<br><br>Mixture of cox confounder adjusted and propensity matched	Very serious<br><br>I^2=95%	Very serious	serious	None	Not reported in all studies/51991	Not reported in all studies/190164	**HR 0.86**<br>(0.7 to 1.06)	⊕◯◯◯<br>Very low

e) Statins compared to no statins and association with incidence of heart failure in patients with atrial fibrillation **(follow-up: 5.7 years)**

1[6]	Non-randomised observational cohort studies	Very Serious<br><br>Cox confounder adjusted	Not serious	serious	serious	None	440/2883	1112/5092	**HR 0.91**<br>(0.80 to 1.03)	⊕◯◯◯<br>Very low

f) Statins compared to no statins and association with incidence of heart failure with preserved ejection fraction in patients with atrial fibrillation **(follow-up: 5 years)**

1[12]	Non-randomised observational cohort studies	Very Serious<br><br>Unadjusted risk ratio	Not serious	Not serious	Very serious	None	13/118	49/434	**RR 0.98**<br>(0.55 to 1.74)	⊕◯◯◯<br>Very low

g) RAS inhibitors compared to no RAS inhibitor and association with incidence of heart failure in patients with atrial fibrillation **(follow-up: range 1 to 5.7 years)**

3[3, 6, 8]	Non-randomised observational cohort studies	Very serious<br><br>Mixture of cox confounder adjusted and unadjusted event numbers	Serious	Serious	Serious	None	Not reported in all studies/ 21251	Not reported in all studies/ 56475	**Not meta-analysed**	⊕◯◯◯<br>Very low

h) RAS inhibitors compared to no RAS inhibitor and association with incidence of heart failure with preserved ejection fraction in patients with atrial fibrillation **(follow-up: 5 years)**

1[12]	Non-randomised observational cohort studies	Serious<br>Cox confounder adjusted	Not Serious	Not Serious	Serious	None	40/398	22/154	**HR 0.63**<br>(0.37 to 1.07)	⊕◯◯◯<br>Very low

i) ACE inhibitors compared to no ACE inhibitor and association with incidence of heart failure in patients with atrial fibrillation **(follow-up: 5.7 years)**

1[6]	non-randomised observational cohort studies	Very Serious<br>Cox confounder adjusted	Not Serious	Serious	Serious	None	558 / 2924	994 / 5051	**HR 1.06**<br>(0.95 to 1.19)	⊕◯◯◯<br>Very low

j) ACE inhibitors compared to no ACE inhibitor and association with incident heart failure hospitalization in patients with atrial fibrillation **(follow-up: 5.3 years)**

1[5]	non-randomised observational cohort studies	Very Serious<br>Cox confounder adjusted	Not Serious	Serious	Not Serious	None	4566 / 15377	23691/105379	**HR 1.24**<br>(1.20 to 1.28)	⊕◯◯◯<br>Very low

k) ARBs compared to no ARB and association with incidence heart failure in patients with atrial fibrillation **(follow-up: 5.7 years)**

1[6]	non-randomised observational cohort studies	Very Serious<br>Cox confounder adjusted	Not Serious	Serious	Serious	None	349 / 1966	1203 / 6009	**HR 1.10**<br>(0.97 to 1.25)	⊕◯◯◯<br>Very low

l) ARBs compared to no ARB and association with incidence heart failure hospitalization in patients with atrial fibrillation **(follow-up: 5.3 years)**

1[5]	non-randomised observational cohort studies	Very Serious<br>Cox confounder adjusted	Not Serious	Serious	Not Serious	None	2039 / 7197	26218 / 113559	**HR 1.16**<br>(1.11 to 1.21)	⊕◯◯◯<br>Very low

m) Beta-blockers compared to no beta-blockers and association with incidence heart failure hospitalization in patients with atrial fibrillation **(follow-up: 1 year)**

1[3]	non-randomised observational cohort studies	Very Serious<br>cox confounder adjusted	Not serious	Serious	Serious	None	Not reported in all studies/ 29838	Not reported in all studies/ 39071	**HR 1.02**<br>(0.99 to 1.05)	⊕◯◯◯<br>Very low

n) Beta-blockers compared to no beta-blockers and association with incidence heart failure in patients with atrial fibrillation **(follow-up: range 5 to 5.7 years)**

2[6, 8]	non-randomised observational cohort studies	Very Serious<br>Mixture of cox confounder adjusted and unadjusted event numbers	Very serious	Serious	Serious	None	1102 / 6306	533 / 2511	**Not meta-analysed**	⊕◯◯◯<br>Very low

o) Beta-blockers compared to no beta-blockers and association with incidence of heart failure with preserved ejection fraction in patients with atrial fibrillation **(follow-up: 5 years)**

1[12]	non-randomised observational cohort studies	Serious<br>Cox confounder adjusted	Not serious	Not Serious	Very Serious	None	22/237	40/315	**HR 0.77**<br>(0.45 to 1.31)	⊕◯◯◯<br>Very low

p) Beta-1 selective beta-blockers compared to no beta-1 selective beta-blockers and association with incidence of heart failure in patients with atrial fibrillation **(follow-up: 5.7 years)**

1[6]	non-randomised observational cohort studies	Very Serious<br>Cox confounder adjusted	Not Serious	Serious	Serious	None	962 / 5544	590 / 2431	**HR 0.96**<br>(0.86 to 1.08)	⊕◯◯◯<br>Very low

q) Beta-1 selective beta-blockers compared to no beta-1 selective beta-blockers and association with incident heart failure hospitalization in patients with atrial fibrillation **(follow-up: 5.3 years)**

1[5]	non-randomised observational cohort studies	Very Serious<br>Cox confounder adjusted	Not Serious	Serious	Not Serious	None	7798 / 35969	20459 / 84787	**HR 0.92**<br>(0.9 to 0.95)	⊕◯◯◯<br>Very low

r) Non-selective beta-blockers compared to no non-selective beta-blockers and association with incidence of heart failure in patients with atrial fibrillation **(follow-up: 5.7 years)**

1[6]	non-randomised observational cohort studies	Very Serious<br>Cox confounder adjusted	Not Serious	Serious	Serious	None	208 / 1407	1344 / 6568	**HR 0.84**<br>(0.72 to 0.98)	⊕◯◯◯<br>Very low

s) Non-selective beta-blockers compared to no non-selective beta-blockers and association with incident heart failure hospitalization in patients with atrial fibrillation **(follow-up: 5.3 years)**

1[5]	non-randomised observational cohort studies	Very Serious<br>Cox confounder adjusted	Not Serious	Serious	Not Serious	None	1536 / 7577	26721 / 113179	**HR 0.93**<br>(0.88 to 0.98)	⊕◯◯◯<br>Very low

t) Calcium channel blockers compared to no Calcium channel blockers and association with incidence of heart failure in patients with atrial fibrillation **(follow-up: 5.7 years)**

1[6]	non-randomised observational cohort studies	Very Serious<br>unadjusted event numbers	Not Serious	Serious	Serious	None	459 / 2821	1093 / 5154	**RR 0.77**<br>(0.70 to 0.85)	⊕◯◯◯<br>Very low

u) Calcium channel blockers compared to no Calcium channel blockers and association with incidence of heart failure with preserved ejection fraction in patients with atrial fibrillation **(follow-up: 5 years)**

1[12]	Non-randomised observational cohort studies	Very Serious<br>Unadjusted risk ratio	Not serious	Not serious	Very serious	None	51 / 423	11 / 129	**RR 1.41**<br>(0.76 to 2.63)	⊕◯◯◯<br>Very low

v) Heart-active calcium channel blockers compared to no heart-active calcium channel blockers and association with incidence of heart failure in patients with atrial fibrillation **(follow-up: 5.7 years)**

1[6]	non-randomised observational cohort studies	Very Serious<br>Cox confounder adjusted	Not Serious	Serious	Serious	None	78 / 458	1474 / 7517	**HR 0.81**<br>(0.64 to 1.03)	⊕◯◯◯<br>Very low

w) Heart-active calcium channel blockers compared to no heart-active calcium channel blockers and association with incident heart failure hospitalization in patients with atrial fibrillation **(follow-up: range 1 to 5.3 years)**

2[3, 5]	non-randomised observational cohort studies	Very Serious<br>Cox confounder adjusted	Very serious	Serious	Serious	None	Not reported in all studies / 10636	Not reported in all studies / 179029	**Not meta-analysed**	⊕◯◯◯<br>Very low

x) Vessel-active calcium channel blockers compared to no vessel-active calcium channel blockers and association with incidence of heart failure in patients with atrial fibrillation **(follow-up: 5.7 years)**

1[6]	non-randomised observational cohort studies	Very Serious<br>Cox confounder adjusted	Not Serious	Serious	Serious	None	393 / 2454	1159 / 5521	**HR 0.76**<br>(0.67 to 0.86)	⊕◯◯◯<br>Very low

y) Vessel-active calcium channel blockers compared to no vessel-active calcium channel blockers and association with incident heart failure hospitalization in patients with atrial fibrillation **(follow-up: 5.3 years)**

1[5]	non-randomised observational cohort studies	Very Serious<br>Cox confounder adjusted	Not Serious	Serious	Not Serious	None	2818 / 10444	25439 / 110312	**HR 1.06**<br>(1.01 to 1.1)	⊕◯◯◯<br>Very low

z) Digoxin compared to no digoxin and association with incidence of heart failure hospitalization in patients with atrial fibrillation **(follow-up: range 1 to 2.8 years)**

2[3, 14]	non-randomised observational cohort studies	Very Serious<br>Mix of propensity matched and cox confounder adjusted	Not serious	Very serious	Serious	None	Not reported in all studies / 10201	Not reported in all studies / 67689	**Not Meta-analysed**	⊕◯◯◯<br>Very low

aa) Digoxin compared to no digoxin and association with incidence of heart failure in patients with atrial fibrillation **(follow-up: 5.7 years)**

1[6]	non-randomised observational cohort studies	Very Serious<br>Cox confounder adjusted	Not Serious	Serious	Serious	None	581 / 2381	971 / 5594	**HR 1.03**<br>(0.92 to 1.15)	⊕◯◯◯<br>Very low

bb) Diuretics compared to no diuretics and association with incident heart failure hospitalization in patients with atrial fibrillation **(follow-up: 1 year)**

1[3]	non-randomised observational cohort studies	Very Serious<br>Cox confounder adjusted	Not serious	Serious	Serious	None	Not reported in all studies / 15733	Not reported in all studies / 53176	**HR 3.05**<br>(2.96 to 3.14)	⊕◯◯◯<br>Very low

cc) Diuretics compared to no diuretics and association with incident heart failure in patients with atrial fibrillation **(follow-up: range 5 to 5.7 years)**

2[6, 8]	non-randomised observational cohort studies	Very Serious<br>unadjusted event numbers	Very serious	Serious	Serious	None	1119 / 5030	516 / 3787	**Not meta-analysed**	⊕◯◯◯<br>Very low

dd) Loop diuretics compared to no loop diuretics and association with incidence of heart failure in patients with atrial fibrillation **(follow-up: 5.7 years)**

1[6]	non-randomised observational cohort studies	Very Serious<br>Cox confounder adjusted	Not Serious	Serious	Serious	None	867 / 3225	685 / 4750	**HR 1.41**<br>(1.25 to 1.57)	⊕◯◯◯<br>Very low

ee) Loop diuretics compared to no loop diuretics and association with incident heart failure hospitalization in patients with atrial fibrillation **(follow-up: 5.3 years)**

1[5]	non-randomised observational cohort studies	Very Serious<br>Cox confounder adjusted	Not Serious	Serious	Not Serious	None	2137 / 7524	26120 / 113232	**HR 1.28**<br>(1.23 to 1.34)	⊕◯◯◯<br>Very low

ff) Thiazides compared to no thiazides and association with incidence of heart failure in patients with atrial fibrillation **(follow-up: 5.7 years)**

1[6]	non-randomised observational cohort studies	Very Serious<br>Cox confounder adjusted	Not Serious	Serious	Serious	None	387 / 2454	1165 / 5521	**HR 0.74** (0.65 to 0.84)	⊕◯◯◯ Very low

gg) Thiazides compared to no thiazides and association with incident heart failure hospitalization in patients with atrial fibrillation **(follow-up: 5.3 years)**

1[5]	non-randomised observational cohort studies	Very Serious<br>Cox confounder adjusted	Not Serious	Serious	Not Serious	None	1455 / 6004	26802 / 114752	**HR 0.98** (0.93 to 1.03)	⊕◯◯◯ Very low

hh) Mineralocorticoid inhibitors compared to no mineralocorticoid inhibitors and association with incidence of heart failure in patients with atrial fibrillation **(follow-up: 5.7 years)**

1[6]	non-randomised observational cohort studies	Very Serious<br>Cox confounder adjusted	Not Serious	Serious	Serious	None	193/715	1359/7260	**HR 1.22** (1.04 to 1.44)	⊕◯◯◯ Very low

ii) Mineralocorticoid inhibitors compared to no mineralocorticoid inhibitors and association with incident heart failure hospitalization in patients with atrial fibrillation **(follow-up: 5.3 years)**

1[5]	non-randomised observational cohort studies	Very Serious<br>Cox confounder adjusted	Not Serious	Serious	Not Serious	None	686 / 1992	27571 / 118764	**HR 1.39** (1.28 to 1.50)	⊕◯◯◯ Very low

jj) Oral anticoagulants compared to no oral anticoagulants and association with incident heart failure hospitalization in patients with atrial fibrillation **(follow-up: 1 year)**

1[3]	non-randomised observational cohort studies	Very Serious<br>Cox confounder adjusted	Not serious	Serious	Serious	None	Not reported in all studies / 27360	Not reported in all studies / 41549	**HR 1.00**<br>(0.97 to 1.03)	⊕◯◯◯<br>Very low

kk) Oral anticoagulants compared to no oral anticoagulants and association with incidence of heart failure with preserved ejection fraction in patients with atrial fibrillation **(follow-up: 5 years)**

1[12]	Non-randomised observational cohort studies	Very Serious<br>Unadjusted risk ratio	Not serious	Not serious	Very serious	None	10 / 81	52 / 471	**RR 1.12**<br>(0.59 to 2.11)	⊕◯◯◯<br>Very low

ll) Oral anticoagulants compared to no oral anticoagulants and association with incidence of heart failure in patients with atrial fibrillation **(follow-up: 5 years)**

1[8]	non-randomised observational cohort studies	Very Serious<br>unadjusted event numbers	Not serious	Serious	Serious	None	18 / 153	65 / 689	**RR 1.25**<br>(0.76 to 2.04)	⊕◯◯◯<br>Very low

ACEi: angiotensin-converting enzyme inhibitors; ARB: angiotensin receptor blocker; CI: Confidence interval; HR: hazard ratio; RAS: Renin-angiotensin system; SGLT2 : sodium-glucose cotransporter 2;

a) Statins

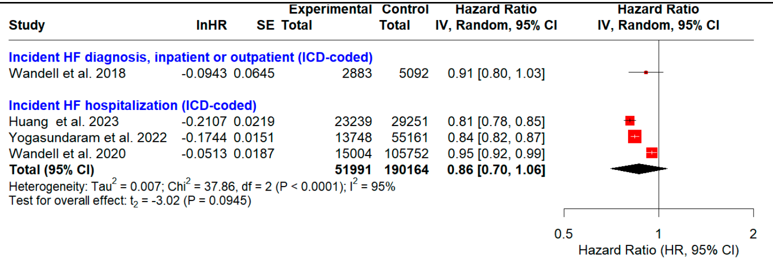

b) Renin-angiotensin system inhibitors

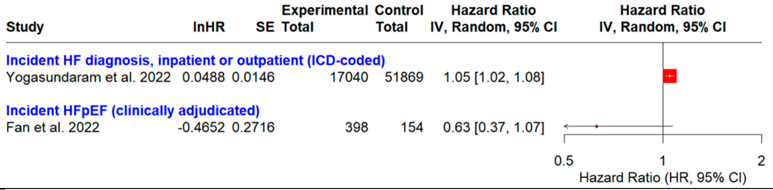

c) Angiotensin-converting enzyme inhibitors

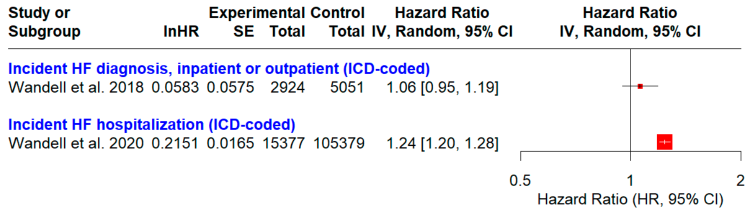

d) Angiotensin receptor blockers

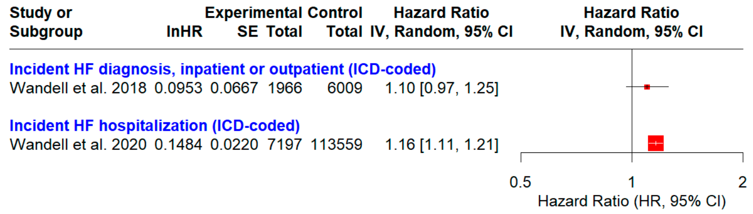

e) Sodium-glucose cotransporter 2 inhibitors

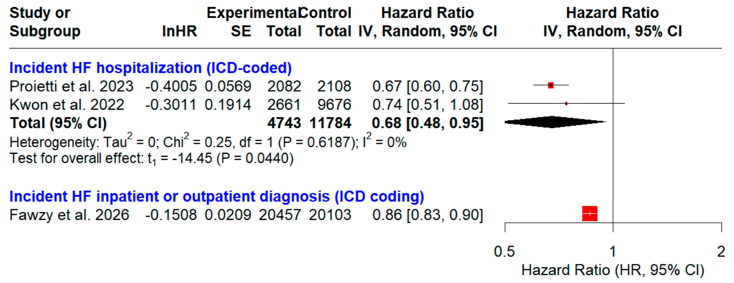

f) Beta-blockers

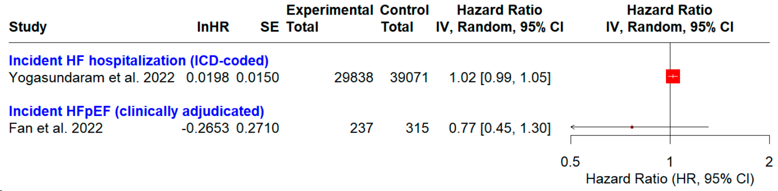

g) Beta-1-selective beta-blockers

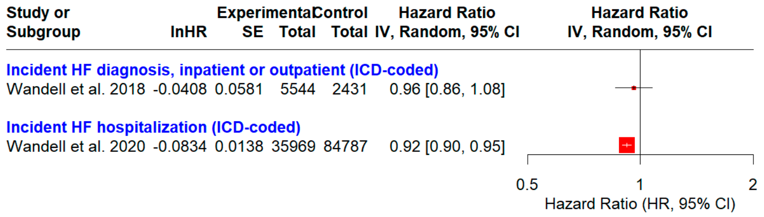

h) Non-selective beta-blockers

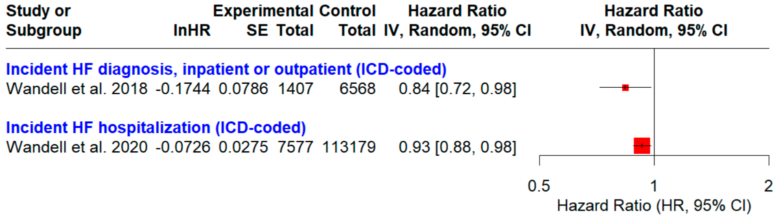

i) Heart-active calcium channel blockers

Study or Subgroup	lnHR	SE	Experimental Total	Control Total	Hazard Ratio IV, 95% CI
**Incident HF diagnosis, inpatient or outpatient (ICD-coded)**					
Wandell et al. 2018	-0.2107	0.1214	458	7517	0.81 [0.64, 1.03]
**Incident HF hospitalization (ICD-coded)**					
Wandell et al. 2020	0.1133	0.0457	1932	118824	1.12 [1.02, 1.22]
Yogasundaram et al. 2022	-0.0834	0.0195	8704	60205	0.92 [0.89, 0.96]

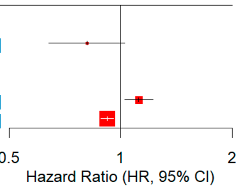

j) Vessel-active calcium channel blockers

Study or Subgroup	lnHR	SE	Experimental Total	Control Total	Hazard Ratio IV, Random, 95% CI
**Incident HF diagnosis, inpatient or outpatient (ICD-coded)**					
Wandell et al. 2018	-0.2744	0.0637	2454	5521	0.76 [0.67, 0.86]
**Incident HF hospitalization (ICD-coded)**					
Wandell et al. 2020	0.0583	0.0218	10444	110312	1.06 [1.02, 1.11]

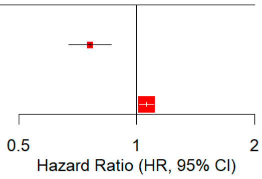

k) Digoxin

Study or Subgroup	lnHR	SE	Experimental Total	Control Total	Hazard Ratio IV, Random, 95% CI
**Incident HF diagnosis, inpatient or outpatient (ICD-coded)**					
Wandell et al. 2018	0.0296	0.0569	2381	5594	1.03 [0.92, 1.15]
**Incident HF hospitalization (ICD-Coded)**					
Yogasundaram et al. 2022	0.4055	0.0170	8376	60533	1.50 [1.45, 1.55]
**Incident HF hospitalization (Trial-cohort)**					
Eisen et al. 2017	0.1655	0.1292	1825	7156	1.18 [0.92, 1.52]

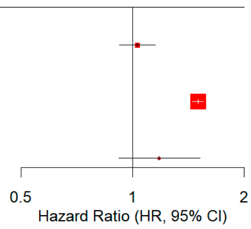

l) Diuretics

Study	lnHR	SE	Experimental Total	Control Total	Hazard Ratio IV, Random, 95% CI
**Incident HF hospitalization (ICD-coded)**					
Yogasundaram et al. 2022	1.1151	0.0150	15733	53176	3.05 [2.96, 3.14]

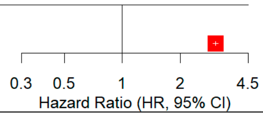

m) Loop diuretics

Study or Subgroup	lnHR	SE	Experimental Total	Control Total	Hazard Ratio IV, Random, 95% CI
**Incident HF diagnosis, inpatient or outpatient (ICD-coded)**					
Wandell et al. 2018	0.3436	0.0581	3225	4750	1.41 [1.26, 1.58]
**Incident HF hospitalization (ICD-coded)**					
Wandell et al. 2020	0.2469	0.0219	7524	113232	1.28 [1.23, 1.34]

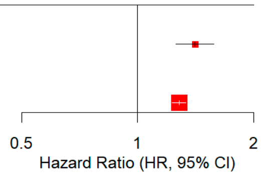

n) Mineralocorticoid inhibitors

Study or Subgroup	lnHR	SE	Experimental Total	Control Total	Hazard Ratio IV, Random, 95% CI
**Incident HF diagnosis, inpatient or outpatient (ICD-coded)**					
Wandell et al. 2018	0.1989	0.0830	715	7260	1.22 [1.04, 1.44]
**Incident HF hospitalization (ICD-coded)**					
Wandell et al. 2020	0.3293	0.0405	1992	118764	1.39 [1.28, 1.50]

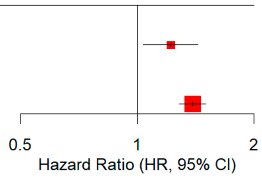

o) Thiazides

Study or Subgroup	lnHR	SE	Experimental Total	Control Total	Hazard Ratio IV, Random, 95% CI
**Incident HF diagnosis, inpatient or outpatient (ICD-coded)**					
Wandell et al. 2018	-0.3011	0.0654	2454	5521	0.74 [0.65, 0.84]
**Incident HF hospitalization (ICD-coded)**					
Wandell et al. 2020	-0.0202	0.0261	6004	114752	0.98 [0.93, 1.03]

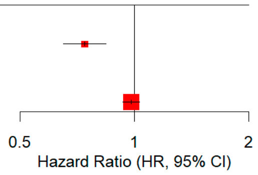

p) Oral anticoagulants

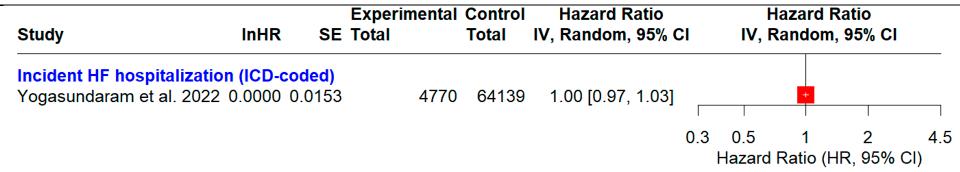

Ln: Natural logarithm; ICD: International Classification of Diseases; HF: Heart Failure; HFpEF: Heart Failure with preserved ejection fraction; HR: Hazard ratio; SE: Standard error

Supplementary Figure S2: Association of drug interventions and new-onset heart failure in patients with atrial fibrillation.

a) Statins

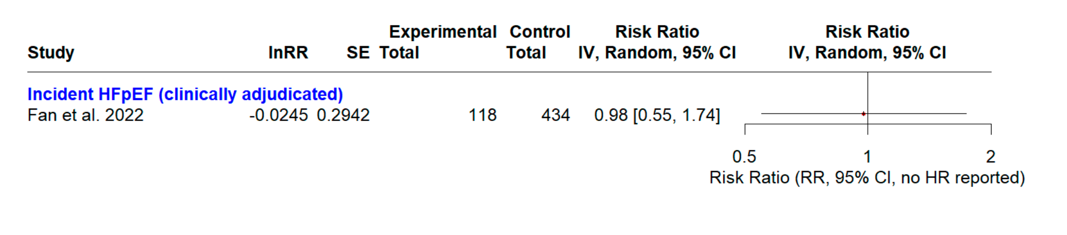

b) Renin-angiotensin system inhibitors

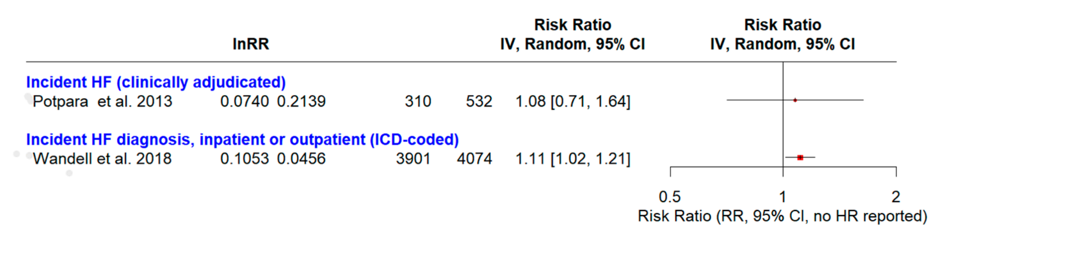

c) Beta-blockers

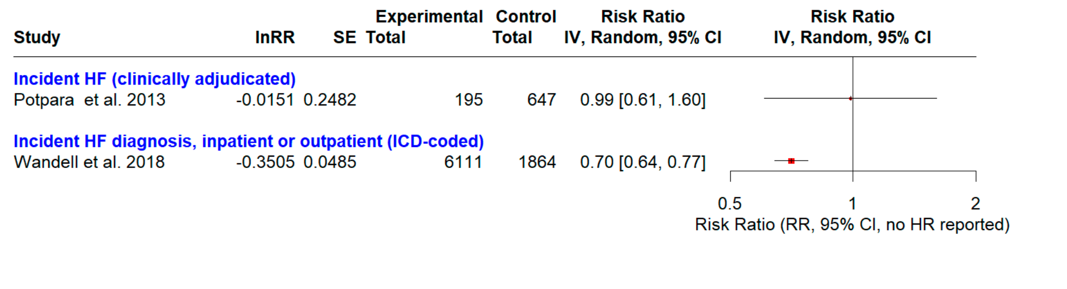

d) Calcium channel blockers

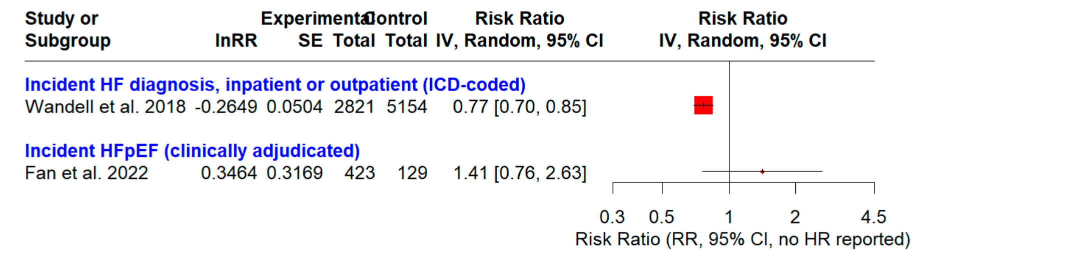

e) Diuretics

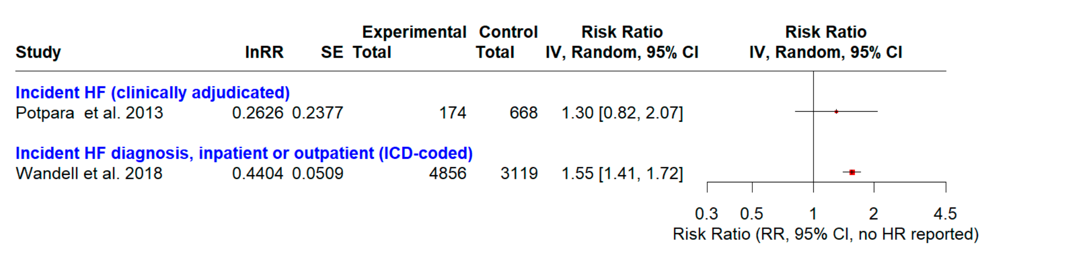

f) Oral anticoagulants

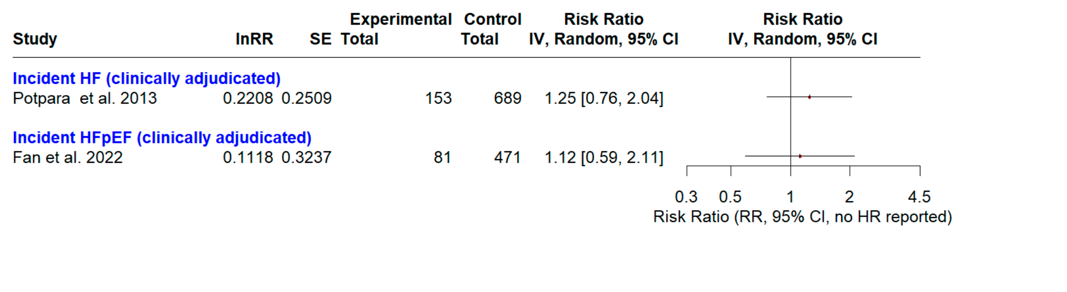

Ln: Natural logarithm; ICD: International Classification of Diseases; HF: Heart Failure; HFpEF: Heart Failure with preserved ejection fraction; RR: Risk ratio; SE: Standard error

Supplementary Figure S3: Risk ratios of incident heart failure in patients with atrial fibrillation summarized by drugs.

Statins	

Study	P-value	Tau2	Tau	I2	HR	95% CI
Omitting Huang et al. 2023	0.3167	0.007	0.085	96%	0.89	[0.41, 1.95]
Omitting Yogasundaram et al. 2022	0.3489	0.012	0.111	97%	0.88	[0.32, 2.42]
Omitting Wandell et al. 2020	0.0599	< 0.001	0.018	46%	0.83	[0.66, 1.04]
**Total (95% CI)**	**0.0945**	**0.007**	**0.0811**	**95%**	**0.86**	**[0.70, 1.06]**

Test for overall effect: $t_2 = -3.02$ (P = 0.0945)

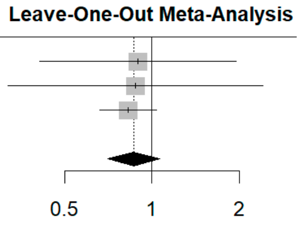

Supplementary Figure S4: Leave one out sensitivity analysis

References:

1. Munn Z, Stone JC, Aromataris E, Klugar M, Sears K, Leonardi-Bee J, et al. Assessing the risk of bias of quantitative analytical studies: introducing the vision for critical appraisal within JBI systematic reviews. JBI Evidence Synthesis. 2023;21(3).

2. Proietti R, Rivera-Caravaca JM, Lopez-Galvez R, Harrison SL, Marin F, Underhill P, et al. Cerebrovascular, Cognitive and Cardiac Benefits of SGLT2 Inhibitors Therapy in Patients with Atrial Fibrillation and Type 2 Diabetes Mellitus: Results from a Global Federated Health Network Analysis. J Clin Med. 2023;12(8). doi: https://dx.doi.org/10.3390/jcm12082814.

3. Yogasundaram H, Islam S, Dover DC, Hawkins NM, Ezekowitz J, Kaul P, et al. Nationwide Study of Sex Differences in Incident Heart Failure in Newly Diagnosed Nonvalvular Atrial Fibrillation. CJC open. 2022;4(8):701–8. doi: https://dx.doi.org/10.1016/j.cjco.2022.04.010.

4. Westergaard LM, Alhakak A, Rorth R, Fosbol EL, Kristensen SL, Svendsen JH, et al. Ventricular rate in atrial fibrillation and the risk of heart failure and death. Europace. 2023;25(5). doi: https://dx.doi.org/10.1093/europace/euad088.

5. Wandell P, Carlsson AC, Li X, Holzmann MJ, Sundquist J, Sundquist K. Use of cardiovascular drugs and risk of incident heart failure in patients with atrial fibrillation. J Clin Hypertens (Greenwich). 2020;22(8):1396–405. doi: https://dx.doi.org/10.1111/jch.13931.

6. Wandell P, Carlsson AC, Holzmann MJ, Arnlov J, Sundquist J, Sundquist K. Associations between relevant cardiovascular pharmacotherapies and incident heart failure in patients with atrial fibrillation: a cohort study in primary care. J Hypertens. 2018;36(9):1929–35. doi: https://dx.doi.org/10.1097/HJH.0000000000001813.

7. Lee K-H, Li S-Y, Liu J-S, Huang C-T, Chen Y-Y, Lin Y-P, et al. Association of warfarin with congestive heart failure and peripheral artery occlusive disease in hemodialysis patients with atrial fibrillation. Journal of the Chinese Medical Association. 2017;80(5):277–82. doi: https://dx.doi.org/10.1016/j.jcma.2016.10.012.

8. Potpara TS, Polovina MM, Licina MM, Marinkovic JM, Lip GYH. Predictors and prognostic implications of incident heart failure following the first diagnosis of atrial fibrillation in patients with structurally normal hearts: the Belgrade Atrial Fibrillation Study. Eur J Heart Fail. 2013;15(4):415–24. doi: https://dx.doi.org/10.1093/eurjhf/hft004.

9. Gao S, Dai W, Zhang L, Juhaeri J, Wang Y, Caubel P. Risk of Cardiovascular Events, Stroke, Congestive Heart Failure, Interstitial Lung Disease, and Acute Liver Injury: Dronedarone versus Amiodarone and Other Antiarrhythmics. J Atr Fibrillation. 2013;6(4):890. doi: https://dx.doi.org/10.4022/jafib.890.

10. Krisai P, Johnson LSB, Moschovitis G, Benz A, Ramasundarahettige C, McIntyre WF, et al. Incidence and Predictors of Heart Failure in Patients With Atrial Fibrillation. CJC Open. 2021;3(12):1482–9. doi: 10.1016/j.cjco.2021.07.016.

11. Huang J-Y, Chan Y-H, Tse Y-K, Yu S-Y, Li H-L, Chen C, et al. Statin Therapy Is Associated With a Lower Risk of Heart Failure in Patients With Atrial Fibrillation: A Population-Based Study. J Am Heart Assoc. 2023;12(23):e032378. doi: https://dx.doi.org/10.1161/JAHA.123.032378.

12. Fan L, Pan J-A, Lin H, Wang C-Q, Zhang J-F, Gu J. Optimal management of blood glucose, blood pressure and atrial fibrillation to reduce the risk of heart failure with preserved ejection fraction. Intern Med J. 2022;52(2):301–9. doi: https://dx.doi.org/10.1111/imj.15006.

13. Kwon CH, Kim YJ, Kim MJ, Cha MJ, Cho MS, Nam GB, et al. Effect of Sodium-Glucose Cotransporter Inhibitors on Major Adverse Cardiovascular Events and Hospitalization for Heart Failure in Patients With Type 2 Diabetes Mellitus and Atrial Fibrillation. Am J Cardiol. 2022;178:35–42. doi: 10.1016/j.amjcard.2022.05.017.

14. Eisen A, Ruff CT, Braunwald E, Hamershock RA, Lewis BS, Hassager C, et al. Digoxin Use and Subsequent Clinical Outcomes in Patients With Atrial Fibrillation With or Without Heart Failure in the ENGAGE AF-TIMI 48 Trial. J Am Heart Assoc.6(7):e006035. doi: 10.1161/JAHA.117.006035.

15. Lin S-M, Liu PP-S, Tu Y-K, Lai EC-C, Yeh J-I, Hsu J-Y, et al. Risk of heart failure in elderly patients with atrial fibrillation and diabetes taking different oral anticoagulants: a nationwide cohort study. Cardiovasc Diabetol. 2023;22(1):1. doi: 10.1186/s12933-022-01688-1.

16. Fawzy A, Bisson A, Fauchier L, Lip G. Impact of sodium-glucose cotransporter 2 inhibitors on bleeding, hospitalizations, and other adverse outcomes in atrial fibrillation patients with and without diabetes. Pol Arch Intern Med. 2026;136(3):17227. doi: 10.20452/pamw.17227.

17. Kipp R, Askari M, Fan J, Field Michael E, Turakhia Mintu P. Real-World Comparison of Classes IC and III Antiarrhythmic Drugs as an Initial Rhythm Control Strategy in Newly Diagnosed Atrial Fibrillation. JACC: Clinical Electrophysiology. 2019;5(2):231–41. doi: 10.1016/j.jacep.2018.08.025.

18. Yusuf S, Healey JS, Pogue J, Chrolavicius S, Flather M, Hart RG, et al. Irbesartan in patients with atrial fibrillation. N Engl J Med. 2011;364(10):928–38. doi: 10.1056/NEJMoa1008816.
